# Molecular phylogenetics of the African horseshoe bats (Chiroptera: Rhinolophidae): expanded geographic and taxonomic sampling of the Afrotropics

**DOI:** 10.1186/s12862-019-1485-1

**Published:** 2019-08-22

**Authors:** Terrence C. Demos, Paul W. Webala, Steven M. Goodman, Julian C. Kerbis Peterhans, Michael Bartonjo, Bruce D. Patterson

**Affiliations:** 1 Integrative Research Center, Field Museum of Natural History, Chicago, IL 60605 USA; 2 Department of Forestry and Wildlife Management, Maasai Mara University, Narok, Kenya; 3 Association Vahatra, BP 3972, 101 Antananarivo, Madagascar; 4 College of Professional Studies, Roosevelt University, Chicago, IL 60605 USA; 5 Mammalogy Section, National Museums of Kenya, Nairobi, Kenya

**Keywords:** Afrotropical biodiversity, East Africa, Introgression, Introns, Phylogeny, *Rhinolophus*, Species tree, Taxonomy

## Abstract

**Background:**

The Old World insectivorous bat genus *Rhinolophus* is highly speciose. Over the last 15 years, the number of its recognized species has grown from 77 to 106, but knowledge of their interrelationships has not kept pace. Species limits and phylogenetic relationships of this morphologically conservative group remain problematic due both to poor sampling across the Afrotropics and to repeated instances of mitochondrial-nuclear discordance. Recent intensive surveys in East Africa and neighboring regions, coupled with parallel studies by others in West Africa and in Southern Africa, offer a new basis for understanding its evolutionary history.

**Results:**

We investigated phylogenetic relationships and intraspecific genetic variation in the Afro-Palearctic clade of Rhinolophidae using broad sampling. We sequenced mitochondrial cytochrome-*b* (1140 bp) and four independent and informative nuclear introns (2611 bp) for 213 individuals and incorporated sequence data from 210 additional individuals on GenBank that together represent 24 of the 33 currently recognized Afrotropical *Rhinolophus* species. We addressed the widespread occurrence of mito-nuclear discordance in *Rhinolophus* by inferring concatenated and species tree phylogenies using only the nuclear data. Well resolved mitochondrial, concatenated nuclear, and species trees revealed phylogenetic relationships and population structure of the Afrotropical species and species groups.

**Conclusions:**

Multiple well-supported and deeply divergent lineages were resolved in each of the six African *Rhinolophus* species groups analyzed, suggesting as many as 12 undescribed cryptic species; these include several instances of sympatry among close relatives. Coalescent lineage delimitation offered support for new undescribed lineages in four of the six African groups in this study. On the other hand, two to five currently recognized species may be invalid based on combined mitochondrial and/or nuclear phylogenetic analyses. Validation of these cryptic lineages as species and formal relegation of current names to synonymy will require integrative taxonomic assessments involving morphology, ecology, acoustics, distribution, and behavior. The resulting phylogenetic framework offers a powerful basis for addressing questions regarding their ecology and evolution.

**Electronic supplementary material:**

The online version of this article (10.1186/s12862-019-1485-1) contains supplementary material, which is available to authorized users.

## Background

We remain in an era of biological discovery [1], even for supposedly well-known vertebrate groups such as mammals. In the last 15 years alone, the total number of mammal species has grown by fully 20%, while the accumulation of new bat species (26.4%), especially in tropical regions, has grown even faster [2, 3]. The discovery of new bat species in the Afrotropics (Africa south of the Sahara, including Madagascar and continental shelf islands) has paralleled these global trends, buoyed by continuing geographic and taxonomic surveys of bats across the region, a growing number of systematic investigations using molecular phylogenetic and integrative taxonomic approaches, and the use of more powerful and objective means of assessing species boundaries. The species limits of morphologically conservative or cryptic lineages of bats have been greatly clarified by an integrative approach using multi-locus genetic delimitation methods as a starting point for identifying candidate species and then testing them using additional, corroborative data from behavioral, morphological, distributional, and/or ecological information ([4], cf. [5]). New species have also come to light via collecting in previously unsampled regions and through genetic analysis of ancient DNA using new methods [6–9].

The genus *Rhinolophus* offers an instructive example. The sole living genus of the Paleotropical (and southern Palearctic) family Rhinolophidae, *Rhinolophus* is the second-most speciose genus of bat (after *Myotis*). Over the last 15 years, the number of its recognized species has grown from 77 (24 of them Afrotropical; [10]) to 106 (with 33 Afrotropical; [11, 12], an enormous 38% increase. In this study, recent intensive surveys in East Africa and neighboring regions of Africa, coupled with parallel studies by others in West Africa and in Southern Africa, permit a new region-wide multi-locus phylogenetic study of the genus*.*.

**Fig. 1:**
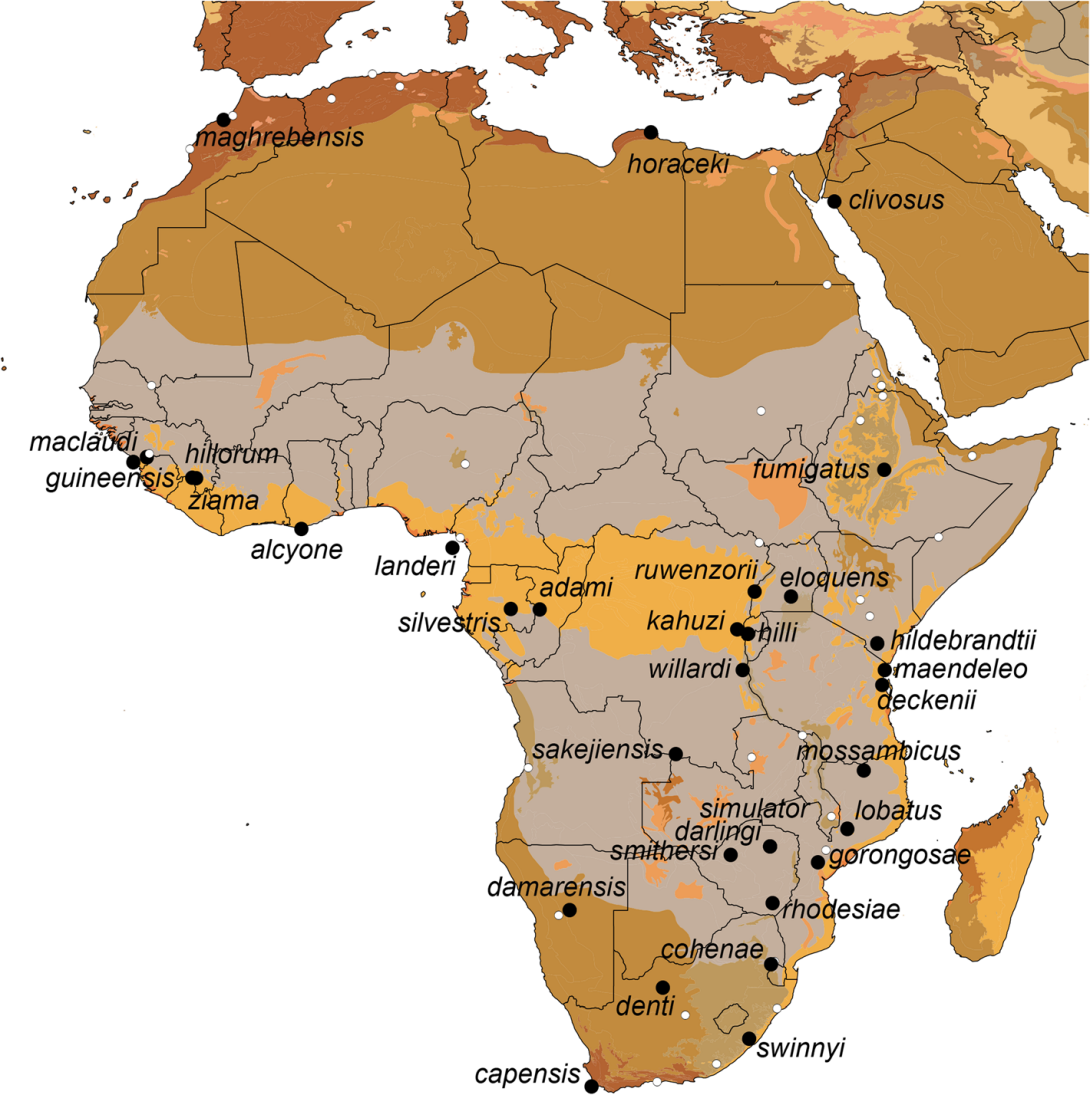
Type localities for recognized species of *Rhinolophus* (black circles), as well as subspecies and synonyms (white circles); label names represent the specific epithets of currently recognized species. Biomes of Africa and neighboring regions indicated by color shading, dark yellow: Tropical and subtropical moist broadleaf forests; orange: Flooded grasslands and savannas; gray: Tropical and subtropical grasslands, savannas, and shrublands; olive brown: Deserts and xeric shrublands; gray-green: Tropical and subtropical moist broadleaf forests; peach: Mangroves; ochre: Mediterranean forests, woodlands, and shrub; dark tan: Tropical and subtropical dry broadleaf forests [14]

Rhinolophidae has been arranged taxonomically on the basis of molecular and morphological data into 5 subgenera by Csorba et al. [13]. Of these 5 subgenera, the subgenus *Rhinolophus* is restricted to Africa and the Palearctic; it includes 7 species groups whose names represent the nomenclatural framework for this study: (1) *R. landeri* group (*landeri*, *alcyone*, *guineensis*, *lobatus*); (2) *R. euryale* group (*euryale*, *blasii*, *mehelyi*); (3) *R. capensis* group (*capensis*, *denti*, *gorongosae, rhodesiae, simulator*, *swinnyi*); (4) *R. adami* group (*adami*, *maendeleo*); (5) *R. ferrumequinum* group (*ferrumequinum*, *bocharicus*, *clivosus*, *damarensis, deckenii*, *hillorum, horaceki*, *nippon*, *sakejiensis*, *silvestris*); (6) *R. maclaudi* group (*maclaudi*, *hilli*, *kahuzi*, *ruwenzorii*, *willardi*, *ziama*); (7) *R. fumigatus* group (*cohenae*, *fumigatus*, *darlingi*, *eloquens*, *hildebrandtii*, *mabuensis*, *mossambicus*, *smithersi*). Of the 33 currently recognized Afrotropical *Rhinolophus* species [10, 11]*,* our study includes at least 24 named taxa (Fig. 1). Multiple well-supported and deeply diverged clades are also revealed by our phylogenetic analyses.

Coalescent species delimitation methods incorporate phylogenetic uncertainty in gene trees and jointly infer species limits and species phylogenies. They have been shown to be conservative in that high delimitation posterior probabilities are consistent indicators of species status ([15] and references therein). Briefly, methods such as the software BPP [15, 16] infer statistical support for genetic isolation on an evolutionary timescale. However, species delimitation based exclusively on molecular data is controversial. It has been shown that multispecies multilocus coalescent delimitation methods can confound species-level and population-level processes and delimit population structure rather than species when the speciation process is protracted ([17] but see also, [18, 19]). However these debates on the status of delimited lineages are resolved, the multispecies coalescent remains a powerful method for inferring the evolutionary independence of lineages that can be subsequently tested with independent data (e.g., morphology, and bioacoustics data in bats) to assess species status in an integrative taxonomy [4]. In this study we carry out lineage delimitation as a foundation for subsequent taxonomic revisions (see [20]). We do not claim that lineages distinguished by our analyses substantiate the boundaries of species; for this reason, we do not formally name these delimited lineages pending integrative taxonomic revision.

The goals of this study are to identify evolutionary lineages among the Afrotropical *Rhinolophus* and to assess their phylogenetic relationships. Lineages for which we were unable to assign confident names (here considered putative species) are considered hypotheses for later testing via integrative taxonomy. More than half of the sequence data used in this study are newly generated, extending the multi-locus analysis of Dool et al. [6] with substantial new material obtained in bat surveys of western, central, eastern, and southern Africa. Our expanded geographic, taxonomic, and population level sampling enables a more robust assessment of phylogenetic relationships and population structure among Afrotropical *Rhinolophus*. The intron data set used here has strong advantages over using mitochondrial loci alone and offers independent representation of the nuclear genome as each of the four introns are found on different chromosomes [6]. Incorporating independent genomic regions into phylogenetic analysis of this monogeneric family [21, 22] and assessment of species relationships and limits is crucial because several instances of mitochondrial introgression have been documented within Rhinolophidae [6, 23–25]. Other instances of possible mitochondrial introgression were investigated via comparisons between our intron phylogenies and those generated with mitochondrial sequences. Finally, using our well resolved nuclear gene tree and species tree, we assess support for broad biogeographic patterns in a comparative context to studies of other Afrotropical bats [13, 20, 22, 26, 27].

## Methods

### Selection of taxa and sampling

**Fig. 2:**
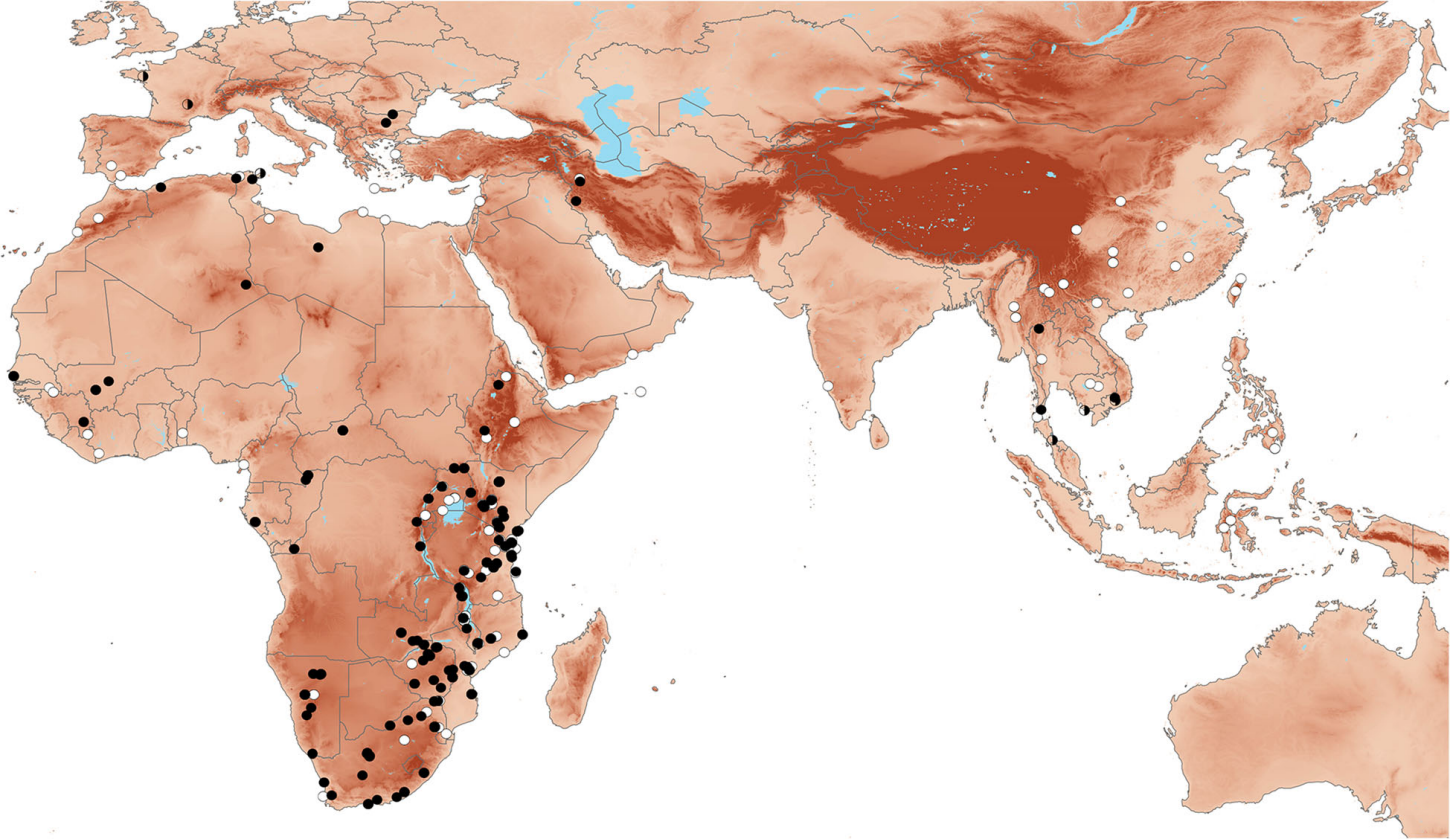
Geographic sampling for the genetic analyses presented by this study. The white circles denote the locations of one or more specimens represented solely by mitochondrial sequences (cyt-*b*), the half-filled circles indicate specimens or groups represented only by nuclear intron sequences, and the black circles identify locations where samples with both mitochondrial and nuclear sequences were acquired

All new genetic data from tissue samples used in this study (*n* = 213) were obtained from specimens previously catalogued and part of the permanent collections of the following natural history museums: Field Museum of Natural History, Chicago, USA; Biodiversity Research and Teaching Collections, Texas A&M University, College Station, USA; Royal Ontario Museum, Toronto, Canada; National Museums of Kenya, Nairobi, Kenya; and Durban Natural Science Museum, Durban, South Africa. No animals were collected in this study; all tissues were parts of permanent research collections. Tissue samples available from Kenya and Tanzania was especially dense (Fig. 2). Initial assignments to species were based on the bats of East Africa key in [26]. An additional 173 cytochrome-*b* (cyt-*b*) sequences and 122 nuclear intron sequences for each of the introns ACOX2, COPS7A, ROGDI, and STAT5A of *Rhinolophus* were downloaded from GenBank from a total of 210 individuals. A species in the recently resurrected genus *Macronycteris* [28], *M. vittatus* (Hipposideridae) was used as an outgroup. In total, 423 individuals with 1–5 genes were analyzed for our study (see Additional file 1 for voucher numbers, locality data, and GenBank accession numbers).

To avoid adding to taxonomic confusion in *Rhinolophus*, we purposefully took a conservative approach to the nomenclatural consequences of our analyses. Where an apparent group’s taxonomic identity is unknown or ambiguous, we refer to it as a numbered clade. This approach was applied to specimens provisionally assigned to combined *Rhinolophus fumigatus* and *R. eloquens* clades that are labeled *fumigatus*/*eloquens*. These names are used as explicit labels for our analysis but cannot vouch for their validity with respect to other taxa that might have nomenclatural priority. Morphological assessment of the clades supported by our analyses will be necessary to determine which existing names can be applied to them.

### Amplification and sequencing

We generally used the methodology previously described by Demos et al. (2018) and Patterson et al. (2018) in the generation and analyses of genetic data for this study. Whole genomic DNA was extracted using the Wizard SV 96 Genomic DNA Purification System (Promega Corporation, WI, USA). Specimens were sequenced for mitochondrial cytochrome-*b* (cyt-*b*), using the primer pair LGL 765F and LGL 766R [29, 30], and four unlinked autosomal nuclear introns: ACOX2 intron 3 (ACOX2), COPS7A intron 4 (COPS7A), and ROGDI intron 7 (ACOX2, COPS7A, ROGDI; [31]); and STAT5A intron 16 (STAT5A; [32]; Table 1, Supplemental Material). Internal primers were designed for the cyt-*b* gene to amplify degraded DNA from a museum skin of putative *Rhinolophus landeri* from Cameroon, the nearest topotype available from a voucher specimen (Additional file 2). PCR amplifications were carried out using the same thermocycler protocols as in [20]. Amplified products were purified using ExoSAP-IT (Thermo Scientific, MA, USA). Sequencing was performed on an ABI 3100 thermocycler (Applied Biosystems, CA, USA) at the Pritzker Laboratory for Molecular Systematics and Evolution, Field Museum of Natural History (FMNH).

**Table 1:** Prior Schemes (PS) used in pairwise BPP analyses. Prior distributions on *τ* represent two relative divergence depths (deep and shallow) and on *θ* represent two relative mutation rate scaled effective population sizes (large and small)

Prior Scheme (PS)	Effective pop. size	Divergence depth	Gamma distribution for prior
1	Large	Deep	*θ* = Γ [1, 10] and *τ* = Γ [1, 10]
2	Large	Shallow	*θ* = Γ [1, 10] and *τ* = Γ [2, 2000]
3	Small	Shallow	*θ* = Γ [2, 2000] and *τ* = Γ [2, 2000]
4	Small	Deep	*θ* = Γ [2, 2000] and *τ* = Γ [1, 10]

Prior distributions on *τ* represent two relative divergence depths (deep and shallow) and on *θ* represent two relative mutation-rate-scaled effective population sizes (large and small)

Sequences were assembled and edited using GENEIOUS PRO v.11.1.5 (Biomatters Ltd.). Sequences were aligned using MUSCLE [33] with default settings in GENEIOUS. Protein coding data from cyt-*b* were translated to amino acids to set codon positions and confirm the absence of premature stop codons, deletions, and insertions. Several gaps were incorporated in the alignments of the nuclear introns, but their positions were unambiguous.

### Gene trees, species trees, and summary statistics

jMODELTEST2 [34] on CIPRES Science Gateway v.3.1 [35] was used to determine the sequence substitution models that best fit the data using the Bayesian Information Criterion (BIC) for cyt-*b* and the four nuclear introns. Uncorrected sequence divergences (*p-*distances) between and within species/clades were calculated for cyt*-b* using MEGA X 10.0.5 [36].

Maximum likelihood estimates of cyt-*b* gene trees and a concatenated alignment of the four partitioned introns were made using the program IQ-TREE version 1.6.0 [37] on the CIPRES portal. We conducted analyses using the ultrafast bootstrap algorithm to search for the best-scoring ML tree algorithm [38] with 1000 bootstrap and 1000 topology replicates. Bayesian gene-tree analyses were carried out in MRBAYES v.3.2.6 [39] on the CIPRES portal to infer individual gene trees for cyt-*b*, the four individual nuclear introns, and the concatenated partitioned alignment of four nuclear introns. Two replicates were run to assist proper mixing. Nucleotide substitution models were unlinked across partitions and then allowed to evolve at individual rates for each locus in the concatenated alignment. Four Markov chains with default heating values were run for 1 × 10^7^ generations and sampled every 1000th generation. Stationarity of MRBAYES results was assessed using TRACER v.1.7 [40]. Majority-rule consensus trees were inferred for each Bayesian analysis.

African taxa assigned to species or clades and named based on support for such clades in the Bayesian and ML analyses of the cyt-*b* and nuclear intron datasets. Thus, results from gene-tree analyses were used to define populations to be used as ‘candidate species’ (as in [41]) in a coalescent-based species-tree approach implemented in StarBEAST2 [42], an extension of BEAST v.2.5.1 [43, 44]. Species-tree analysis was conducted using the four nuclear intron alignments. Substitution, clock, and tree models were unlinked across all loci. A lognormal relaxed-clock model was applied to each locus with a Yule tree prior and a linear with constant root population size model. Analyses were replicated four times with random starting seeds and chain lengths of 2 × 10^8^ generations, with parameters sampled every 20,000 steps. For the StarBEAST2 analyses, evidence for convergence and stationarity of the posterior distribution of model parameters was assessed based on ESS values > 200 and examination of trace files in Tracer v.1.7. Burn-in was set at 20%, and separate runs were assembled using LOGCOMBINER v.2.5.1 and TREEANNOTATOR v.2.5.1 [44].

### Coalescent lineage delimitation

We conducted joint independent lineage delimitation and species-tree estimation using the program BPP v.3.3 [15, 45]. This analysis was carried out to guide future investigations of the lineages inferred here, using an integrative species taxonomic approach to include fixed differences in phenotypic characters, acoustics, ectoparasitic associations, and geographic distributions. BPP analyses were carried on those populations obtained from the concatenated gene-tree analyses and were identical to specimens assigned to lineages in the species-tree analyses. Each population was designated as a putative independent lineage to be evaluated under the multi-species coalescent model [14 and references therein]. Separate analyses were carried out for lineages within each of four different *Rhinolophus* species groups: *capensis* group, six lineages; *ferrumequinum* group, six lineages; *fumigatus/eloquens* group, eight lineages; and *landeri* group, four lineages. The validity of our assignment of specimens to populations was tested using the guide-tree-free algorithm (A11) in BPP. Two replicates were run for each of four different combinations of priors on divergence depth and effective population sizes (*τ* and *θ*, respectively; Table 1), as the probability of delimitation by BPP is sensitive to these two parameters [16, 46]. All BPP analyses were run for 5 × 10^4^ generations, with a burn-in of 10^4^ generations and samples drawn every 50th generation. In total, eight BPP runs were carried out for each of the aforementioned species groups using nuclear intron loci (*n* = 4). Lineages were considered to be statistically well supported when the delimitation posterior probabilities generated were ≥ 0.95 under all four prior combinations.

All newly generated sequences were deposited in GenBank with accession numbers MN025547– MN026153; (see also Additional file 1). Sequence alignments used in this study have been made available on the Figshare data repository (DOI: 10.6084/m9.figshare.8239760).

## Results

The alignment of 351 cyt-*b* sequences used in the ML and BI gene-tree analyses had a total number of base pairs (bp) ranging from 497 to 1140, and averaged 93% coverage of the complete cyt-*b* gene (1140 bp). To aid in visualizing the phylogenies inferred from this matrix, we reduced a matrix of 387 individuals to a set of mostly unique sequences, resulting in a final alignment of 351 individuals. The number of base pairs for the sequence alignments used in individual ML and BI gene trees and Bayesian species tree analyses were: ACOX2 (*n* = 220 ML and BI, 219 species tree), 420–552 bp; COPS7A (*n* = 220 ML and BI, 219 species tree), 581–760 bp; ROGDI (*n* = 219 ML and BI, 217 species tree), 356–500 bp; STAT5A (*n* = 219 ML and BI, 217 species tree), 329–761 bp; and 4 intron concatenated alignment (*n* = 221), 1509–2429 bp. The best supported substitution models for each locus estimated by jMODELTEST2 were: 351 sequence cyt-*b* = GTR + I + G; ACOX2 = K80 + G; COPS7A = HKY + I; ROGDI and STAT5A = HKY + G. Uncorrected cyt-*b p*-distances for African *Rhinolophus* in the 316 sequence cyt-*b* alignment (removing Eurasian sequences except for *R. hipposideros* and *R. xinanzhongguoensis*) ranged from 0.010 to 0.152 between species/clades, while within species/clade distances ranged from 0.000 to 0.025 (Additional file 3).

### Mitochondrial gene trees

**Fig. 3:**
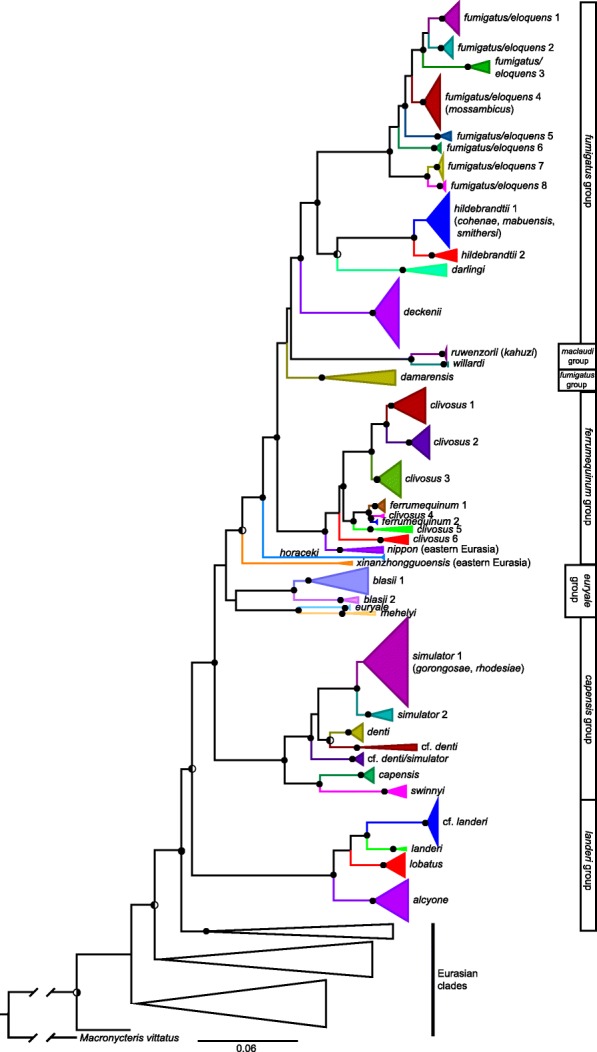
Maximum likelihood phylogeny of mitochondrial cytochrome-*b* sequences of *Rhinolophus*. The phylogeny was inferred in IQ-TREE and its topology was very similar to the Bayesian phylogeny calculated in MRBAYES. Filled black circles on nodes denote bootstrap values (BS) ≥ 70% and Bayesian posterior probabilities (PP) ≥ 0.95, left-half-filled circles indicate BS ≥ 70% and PP < 0.95, right-half-filled circles indicate BS < 70% and PP ≥ 0.95, and unmarked nodes indicate BS < 70% and PP < 0.95. Specific epithets in parentheses following clade names indicate sequences from specimens used in recent species descriptions that were not supported as monophyletic and are subordinate to other clades and would render them paraphyletic. Branch colors indicate individual clade membership; species groups are from [13]

Maximum likelihood (ML) and Bayesian inference (BI) inferred trees with similar topologies; the ML gene tree is shown for the 351 sequence cyt-*b* alignment of 74 *Rhinolophus* species/clades (Fig. 3; see also Additional file 4 for the phylogeny with all 351 terminals labeled). In the cyt-*b* gene tree, a majority of sub-Saharan taxa were strongly supported as monophyletic (i.e., maximum likelihood bootstrap support [BS] ≥ 70%, Bayesian posterior probability [PP] ≥0.95), with several exceptions detailed here. For sub-Saharan African *Rhinolophus*, there were four major well-supported monophyletic endemic haplogroups: a) the *fumigatus* species group that includes eight *R. fumigatus/eloquens* clades, two *R. hildebrandtii* clades, and *R. darlingi*; b) the *maclaudi* species group that includes *R. ruwenzorii* and *R. willardi*, whose phylogenetic position is unresolved; c) the *capensis* species group that includes two *R. simulator* clades, *R. denti*, *R. capensis*, *R. swinnyi*, and two clades provisionally labeled as cf. *denti*/*simulator* and cf. *simulator* distributed widely south of the Sahara; and d) the *landeri* species group consisting of two *R. landeri* clades, *R. lobatus*, and *R. alcyone*. The phylogenetic position [23] of *R. damarensis*, recently elevated because it rendered *R. darlingi* paraphyletic [47], is uncertain. *Rhinolophus damarensis* as currently known is associated with arid southern African habitats. However, a newly available specimen collected in western Democratic Republic of Congo (DRC; Guineo-Congolian rainforest in [48]) is unexpectedly nested within the R. *damarensis* cyt-*b* clade.

Of the two species groups whose members include both African and Palearctic species, the *ferrumequinum* species group is strongly supported as sister to *fumigatus* + *maclaudi* + *damarensis* while the monophyly and position of the *euryale* species group is poorly supported. Species from eastern Eurasia + Australasia cluster outside of African clades in the cyt-*b* tree with two notable exceptions. First, *R. nippon* [formerly *R. ferrumequinum*; 22]) from eastern Eurasia is strongly supported as sister to four endemic Afrotropical *R. clivosus* clades and two *R. ferrumequinum* + *R. clivosus* clades whose distributions include North Africa, Europe, and the Middle East. Second, eastern Eurasian *R. xinanzhongguoensis* [49] has mixed support as sister to taxa in the *fumigatus*, *maclaudi*, and *ferrumequinum* groups (BS = 86%, PP = 0.77). Finally, within the *euryale* group, *R. blasii* includes a clade distributed in eastern and southern Africa that is sister to a North African + Middle Eastern clade. A majority of the deeper nodes are strongly supported (10 of 13).

Several currently recognized species scarcely differ genetically (~ 1% or less cyt-*b* uncorrected *p-*distances); and render other species paraphyletic (see Additional file 4 for a detailed cyt-*b* tree that depicts all 351 labeled terminal branches). In the *maclaudi* species group, *R. kahuzi* is genetically identical to three sequences of *R. ruwenzorii*. In the *fumigatus* species group, *R. smithersi*, *R. cohenae*, and *R. mabuensis* all differ by < 1% in cyt-*b* from *R. hildebrandtii* clade 1 and, if they are valid, would render that species paraphyletic (cf. [50]). In the *capensis* species group, five newly sequenced *R. gorongosae* specimens and three *R. rhodesiae* specimens differed from *R. simulator* clade 1 by only 1.4 and 1%, respectively, in cyt-*b*. Moreover, they are not reciprocally monophyletic and likewise would render *R. simulator* paraphyletic [cf. 12, where *R. gorongosae* is 7.3% cyt-b distant from *R. simulator*; also see Fig. 1, Supplemental Material]. We resequenced a specimen assigned to *R. landeri* by Taylor et al. [12; DM12953, GenBank MG980682], along with another newly obtained specimen from the same locality in Liberia, and found that they nest deeply within *R. blasii* clade 1 (Fig. 3 and Additional file 4). Finally, a monophyletic clade of three specimens from three separate countries in the Central African Guineo-Congolian rainforest region (cf. *denti*) was unexpectedly inferred as nested within the *capensis* group, otherwise distributed in eastern and southern Africa. All other members of the *capensis* group are considered to be savanna/woodland species [13] with the exception of the subspecies *R. simulator alticolus* (see Discussion).

### Concatenated nuclear gene trees

**Fig. 4:**
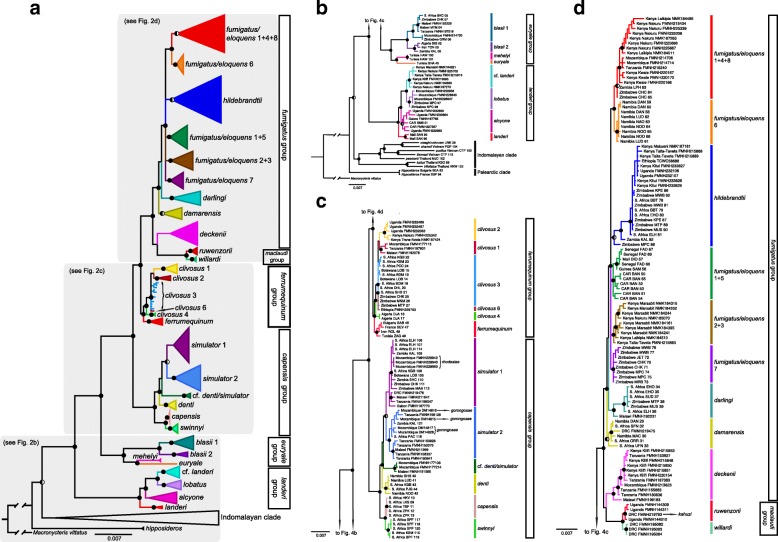
**a** Bayesian phylogeny of *Rhinolophus* based on four nuclear introns. The phylogeny was inferred in MRBAYES and its topology closely resembled the maximum likelihood phylogeny calculated in IQ-TREE. Filled black circles on nodes denote bootstrap values (BS) ≥ 70% and Bayesian posterior probabilities (PP) ≥ 0.95, left-half-filled black circles indicate BS ≥ 70% and PP < 0.95, and unmarked nodes indicate BS < 70% and PP < 0.95. **b**–**d** enlarged sections of the complete nuclear intron tree showing individual relationships. Branch colors indicate individual clade membership; species groups are from [13]. Specimen localities include counties for Kenya. Museum acronyms are defined in Additional file 1

The ML gene tree inferred from concatenation of the nuclear genes ACOX2, COPS7A, ROGDI, and STAT5A (221 individuals; matrix > 99% complete) is shown in Fig. 4 (individual intron gene trees from ML and Bayesian analyses are depicted in Additional file 6). This tree was very similar to the BI tree with most nodes recovered as well supported. Topological differences with the cyt-*b* gene tree exist, including the indeterminate placement of *R. hildebrandtii* within the *fumigatus*/*eloquens* group and strong support for *R. landeri* from Mali as sister to *R.* cf. *landeri* + *R. lobatus* + *R. alcyone*. Incomplete lineage sorting and/or gene flow between recently diverged sisters may account for the lack of monophyly for a) *R. fumigatus/eloquens* clades 1 + 4 + 8, *R. fumigatus/eloquens* clades 1 + 5, *R. fumigatus/eloquens clades* 2 + 3, *R. hildebrandtii* clades 1 + 2, and *R. ferrumequinum* clades 1 + 2.

The remaining clades supported as monophyletic in the cyt-*b* gene tree are moderately or strongly supported as monophyletic in the concatenated nuclear gene tree with the exception of *R. alcyone* which is not supported as monophyletic. *Rhinolophus gorongosae* is not monophyletic and is nested among minimally diverged specimens identified as *R. simulator* by [6; 7 sequences, 21; 1 sequence], and in this study as *R. simulator* 2. This *simulator* clade is distributed in Tanzania, Malawi, Zambia, Mozambique, and South Africa. *Rhinolophus rhodesiae* is likewise nested within *R. simulator* clade 2 that includes sequences from DRC, Botswana, Zambia, Malawi, Mozambique, Zimbabwe, and South Africa. There is no indication of mitochondrial introgression involving either *R. gorongosae* or *R. rhodesiae. Rhinolophus* cf. *denti*/*simulator* is a deeply diverged, monophyletic clade from southeast Africa with uncertain relationships to *R. denti* (southern Africa) and sympatric *R. simulator* 1 + 2. The membership of *R. deckenii* in the *ferrumequinum* group is challenged by its well supported sister relationship to the *fumigatus* group, rather than to *R. clivosus* + *R. ferrumequinum* (Fig. 4). The position of *R. ruwenzorii* and *R. willardi* (*maclaudi* group) is uncertain, although *R. ruwenzorii* + *R. willardi* + *R. deckenii* are strongly supported as sister to members of the *fumigatus* group.

### Species trees

**Fig. 5:**
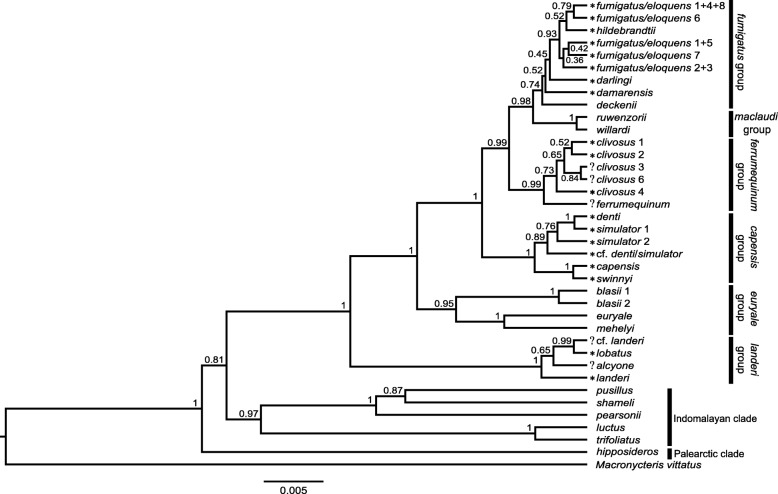
Species tree estimated in StarBEAST2 using the four nuclear intron dataset. Numbers adjacent to nodes indicate posterior probabilities. Terminal tips in the tree that are statistically well-supported (PP ≥ 0.95) from BPP are indicated by “*” preceding the clade name, and terminal tips that had PP < 0.95 are indicated by “?” preceding the clade name. Species groups are from [13]

The four StarBEAST2 runs in the multilocus coalescent species tree analyses converged within 10 × 10^6^ generations. We discarded the first 20% of each run, resulting in 8,000 trees in the posterior distributions. ESS values for all posterior parameters were greater than 300 in the combined species tree analysis of 32,000 trees. The species tree (Fig. 5) is largely in agreement with the concatenated nuclear tree (Fig. 4) in the following respects: a) strongly supports *R. hipposideros* as sister to all other *Rhinolophus* species in the tree; (b) strongly supports the sister relationship of the *R. landeri* group to the remaining African groups in the tree; c) strongly supports *R. landeri* as sister to *R. alcyone* + *R. lobatus* + *R.* cf. *landeri*; and d) strongly supports all the species group assignments made by Csorba et al. 2003, with the exception of *R. deckenii* (which had been uncertainly placed in *ferrumequinum* group) and *R. ruwenzorii* and *R. willardi* (*maclaudi* group), but here recovered in the *fumigatus* group. In contrast to the concatenated analysis, *R. alcyone* is poorly supported as sister to *R. lobatus* + *R.* cf. *landeri* clade. *R. gorongosae* and *R. rhodesiae* are respectively members of well supported monophyletic clades that also include specimens assigned to *simulator* 1 and *simulator* 2 in the cyt-*b* tree (Fig. 3 and Additional file 4), although *R. simulator* clades 1 and 2 in the species tree analysis (Fig. 5) have different memberships than the two clades in the mitochondrial gene tree (Fig. 3) with the same labels. The clades labeled *simulator* 1 and *simulator* 2 in the species tree analysis (Fig. 5) have largely overlapping distributions although *simulator* 1 also includes specimens from DRC and Gabon that were provisionally assigned to *R*. cf. *denti* in the cyt-*b* gene tree (Fig. 3). The enigmatic placement of these Guineo-Congolian rainforest [48] specimens within *R. simulator* 1, otherwise distributed in savanna/woodland, warrants further investigation.

### Lineage delimitation

Results from the replicated BPP analyses show that prior choice had minimal effect on delimitation probabilities for most of the 23 tested species/clades (Table 2).

**Table 2:** Summary of BPP for the four intron dataset. Values for BPP putative species are average posterior probabilities (PP) of delimitation from three replicated BPP runs under each of four different Prior Schemes for four datasets (PS; Table 1). Boldface values indicate overall BPP results with PP ≥ 0.95

Putative species	BPP PS1	BPP PS2	BPP PS3	BPP PS4	BPP Overall
*fumigatus/eloquens* 1 + 4 + 8	1	1	1	1	**1**
*fumigatus/eloquens* 1 + 5	1	1	1	1	**1**
*fumigatus/eloquens* 2 + 3	1	1	1	1	**1**
*fumigatus/eloquens* 6	1	1	1	1	**1**
*fumigatus/eloquens* 7	1	1	1	1	**1**
*hildebrandtii*	1	1	1	1	**1**
*darlingi*	1	1	1	1	**1**
*damarensis*	1	1	1	1	**1**
*clivosus* 1	0.99	0.99	0.99	0.99	**0.99**
*clivosus* 2	0.99	0.99	0.99	0.99	**0.99**
*clivosus* 3	0.94	0.95	0.92	0.93	0.94
*clivosus* 4	0.99	0.99	1	0.99	**1**
*clivosus* 6	0.93	0.94	0.91	0.93	0.93
*simulator* 1	1	1	1	1	**1**
*simulator* 2	1	1	1	1	**1**
*denti*	1	1	1	1	**1**
cf. *denti/simulator*	1	1	1	1	**1**
*capensis*	1	0.99	1	1	**1**
*swinnyi*	1	0.99	1	1	**1**
*landeri*	0.99	0.98	0.99	0.99	**0.99**
cf. *landeri*	0.99	0.99	0.92	0.86	0.94
*lobatus*	0.99	0.99	0.99	0.99	**0.99**
*alcyone*	0.99	0.98	0.92	0.86	0.94

Values for BPP PSs are average posterior probabilities (PP) of delimitation from three replicated BPP runs under each of four different Prior Schemes for two datasets (PS; Table 1). Boldface values indicate overall BPP results with PP ≥ 0.95

However, for the four clades whose mean PP in the four summed Partition Schemes (see Table 1 for prior scheme definitions) fell below a threshold of 0.95, PS 3 and 4 had the most influence. The clades that were not delimited all had PP ≤ 0.95 but ≥0.90 and thus had marginal support. Most of the unsupported clades had short branches and weak node support in the species tree analysis (Fig. 5). Distinguishing robustly defined lineages by congruence across all Prior Schemes, 19 evolutionarily independent lineages are delimited including all six lineages analyzed in the *capensis* group (these include two possibly new species; three of five lineages in the *ferrumequinum* group, including 2 possibly new species; two of four lineages in the *landeri* group, including strong support for recently recognized *R. lobatus* (distinct from *R. landeri* [12] and the newly sequenced *R.* cf. *denti/simulator*; and finally 8 of 8 lineages in the *fumigatus* group, including three possibly new species as well as support for the recent recognition of *R. damarensis* as a valid species [47]. However, there was no PP support for alternative delimitations of clades; that is, all alternate delimitations that statistically tested the merger of two or more putative species had PP ≤ 0.20). The eight strongly delimited clades that could not be confidently named are candidates to be evaluated as potentially valid species using independent datasets.

## Discussion

### Phylogenetic relationships of *Rhinolophus*

This is the broadest phylogenetic study of Afrotropical species in the genus *Rhinolophus* published to date. Multiple phylogenetic studies have confirmed the monophyly of *Rhinolophus* and the Rhinolophidae [20, 51, 52]. Csorba et al. [13] presented a phylogenetic hypothesis for the monophyly of African *Rhinolophus*, with *R. blasii* and *R. clivosus* extending from the Afrotropics to the Western Palearctic, and *R. euryale*, *R. ferrumequinum*, and *R. hipposideros* having distributions in both North Africa and the Western Palearctic. Several studies have placed the most recent common ancestor of *Rhinolophus* at ~ 40 Mya [53, 54]; cf. ([22], at 34 Mya). It has long been considered that Rhinolophidae originated in the African or Asian tropics, although Csorba et al. [13] presented data supporting a European origin of the family when tropical conditions prevailed in southern Europe during the Miocene. However, Dool et al. [6] argued instead for a Middle Eastern origin for the basal lineage *R. hipposideros*. Owing to poor resolution of basal nodes in their multi-locus phylogeny [6], they refrained from speculating on the ancestral origin of Rhinolophidae but, did find support for accelerated diversification within Afrotropical *Rhinolophus* over the last 6 Mya. Subsequently, the new species *R. xinanzhongguoensis*, described from southwestern China [47], was strongly supported as having affinities to the *ferrumequinum*, *fumigatus*, and *maclaudi* groups.

This Eastern Palearctic/Indomalayan species is phylogenetically nested deeply within the African *Rhinolophus* radiation (Fig. 3); it has mixed support (BS = 86%, PP = 0.77) as sister to the African *fumigatus* and *maclaudi* species groups plus the Afro-Palearctic *ferrumequinum* group that includes another Eastern Palearctic species, *R. nippon* [23]. Both of these Eurasian species are closer to the endemic African groups *fumigatus* and *maclaudi* than to the endemic Afrotropical *capensis* and *landeri* groups. Thus, the membership of *R. xinanzhongguoensis* and *R. nippon* in a predominantly African clade seems to indicate a complex historical biogeographical relationship between the Afrotropics and Eastern Eurasia, possibly supporting additional dispersal events between the continents. However, it should be noted that data is still lacking from independent nuclear loci for *R. xinanzhongguoensis* and *R. nippon*.

### Lineage delimitation and taxonomic reappraisal

The phylogenetic relationships of Afrotropical *Rhinolophus* species inferred here are in broad agreement with the study of Dool et al. [6], based on six introns. To extend their findings, we sampled four of their introns for 99 vouchered specimens representing eight monophyletic cyt-*b* clades not present in their study. We also sequenced members of nine clades represented in their study with samples from new Afrotropical localities. Our expanded data set is the largest yet for Afrotropical *Rhinolophus*, and infers support for up to 23 independent evolutionary lineages as candidate species for future assessment with corroborative data. Results from coalescent delimitation and species tree analysis suggest three named species may be synonyms (*R. kahuzi* and either *R. gorongosae* or *R. rhodesiae*). Although we did not have access to tissue samples from *R. smithersi*, *R. cohenae*, and *R. mabuensis*, and so lack intron data, cyt-*b* sequences from GenBank indicate that these recently described taxa are minimally divergent from *R. hildebrandtii* (< 1% in cyt-*b*), and their recognition would render it paraphyletic.

Taylor et al. [12] recently argued for species status for two African *Rhinolophus* names long regarded as synonyms: *R. lobatus* (Peters, 1852) from *R. landeri* and *R. rhodesiae* (Roberts, 1946) from *R. swinnyi*. They described the new species *R. gorongosae* on the basis of integrative data that included a suite of morphological variables, but their molecular phylogenetic analyses relied solely on cytochrome-*b*. Their putative *R. gorongosae* (DM14815, DM14843) had anomalously long branches (*p*-distance 7.3 and 7.2% from *R. simulator* and putative *R. rhodesiae*, respectively; see their Fig. 2) and nested within *R. simulator*, which motivated us to re-examine these deeply diverged specimens. In addition, the sister relationship they determined of *R. landeri* and *R. gorongosae* (15% *p*-distance), instead of with *alcyone and lobatus* [other members of the *landeri* group; 13], led us to compare this sequence to GenBank accessions using BLASTn. The BLASTn query showed 100% identity of their *R. landeri* (GenBank accession MG980682, DM12953, Liberia) with *Bos taurus*. When tissue from this voucher specimen and another (DM12622), also from Liberia, was extracted and new sequence data generated, those individuals were found to nest deeply within the *R. blasii* clade 1 (Additional file 4). We extracted and sequenced five samples identified as *R. gorongosae* from the Durban Museum, including DM14815 from [12], and found them to be 1–1.4% cyt-*b* diverged from *R. simulator* and *R. rhodesiae*. This strongly suggests that the genetic arguments in [12] for the newly described *R. gorongosae* and for elevation of *R. swinnyi rhodesiae* to species rank were based on sequencing error (see Additional file 5 for comparison of GenBank sequences versus newly sequenced material).

Several studies have demonstrated instances of mitochondrial introgression (i.e. mitochondrial capture) among populations of *R. ferrumequinum* and *R. clivosus* [6]; *R. sinicus*, *R. rouxii*, *R. pearsonii pearsonii*, and *R. p. chinensis*, restricted to eastern Eurasia (Mao et al. [24, 25], and the *R. macrotis* species complex in China [55]. Potential mitochondrial introgression is apparent in our study in the cyt-*b* gene tree for *ferrumequinum* clades 1 and 2 and *clivosus* clade 2 as discussed in [6]. Taylor et al. [12] suggested historical genetic introgression might account for the discrepancy between the morphological disparity of *R. simulator* and *R. rhodesiae* and their lack of genetic differentiation (0.6%). However, they did not test this hypothesis with genetic data. In this study, mitochondrial, concatenated nuclear loci, and the species tree (also inferred with nuclear data only) all strongly infer the very close relationship of *R. rhodesiae* to *R. simulator* clade 1. Although the cyt-*b* tree recovers both *gorongosae* and *rhodesiae* as paraphyletic, the concatenated nuclear phylogeny recovers them in two separate monophyletic clades (*simulator* 1 and 2; Fig. 4). To understand these conflicting signals, and to determine which of these clades actually represents true *R. simulator*, a geographically expanded integrative taxonomic assessment will be necessary.

### Range extensions and biogeographic patterns

The broad phylogenetic and geographic sampling in our study uncovered a number of range extensions for described species and also suggests possible niche divergences of putative undescribed species based on their genetic and geographic relationships. In the *capensis* group, a clade from Cameroon (ROM 68963), Gabon (FMNH 167770), and western DRC (FMNH 219476) is strongly supported in the cyt-*b* gene tree (Fig. 3) as sister to *R. denti*, otherwise known from savanna and woodlands of southern (*R. denti*) and western (*R. denti knorri*) Africa. The specimens from DRC and Gabon were included in the concatenated nuclear intron tree and found to nest well within *R. simulator*, which is sister to *R. denti*. *Rhinolophus alcyone alticolus* Sanborn, 1936 [56] was allocated to *R. simulator* by Koopman [57], who was followed by subsequent authors, but Csorba et al. [13] suggested that *R. simulator alticolus* might prove to be a separate species. The type specimen (which is now lost) was from Mt. Cameroon, and western Cameroon is the only lowland rainforest distribution for the savanna woodland *simulator* [13]. The specimen we sequenced from Mt. Cameroon was from a ~ 50-year-old skin and nuclear genes were not successfully amplified. Nonetheless, the strong support for the DRC and Gabon specimens (from the same clade as the Mt. Cameroon specimen in the cyt-*b* tree) with *R. simulator* suggests that our cf. *denti* clade may be introgressed *R. simulator* whose range now extends well into the western African rainforest habitat [48]. Independent nuclear data from additional specimens are needed to confirm the status of this clade. Also within the *capensis* group, all analyses in our study strongly support the existence of an undescribed species provisionally designated cf. *denti/simulator*. At present, populations are known from Tanzania, Mozambique, and Malawi where they are sympatric with its close relative *R. simulator* and presumably differ from it ecologically.

In the *euryale* group, two newly sequenced individuals from Liberia are strongly supported as sisters of populations identified as *R. blasii* populations from southeastern Africa. This extends the range of sub-Saharan populations clade > 5000 km west of their current distribution. However, *R. blasii* has a highly disjunct distribution, and individuals from southern Europe (its type locality is Italy) were not included in our analysis; if conspecific with Moroccan populations of *R. blasii,* the Liberian records document a 1500 km range extension. Both specimens had been identified as *R. landeri*; if mitochondrial introgression was responsible, it is unclear where contact may have occurred. In the *maclaudi* group, the range of *R. ruwenzorii* is now extended to the mountains west of Lake Kivu in Kahuzi-Biega NP, as strong support from mitochondrial and nuclear data indicate that *R. kahuzi* [58] is a synonym of *R. ruwenzorii.*

As in Csorba et al. [13] and Dool et al. [6], the most basal lineage within the African radiation is the *landeri* group (*alcyone*, *landeri*, and *lobatus*), whose partial distribution in rainforest habitats has been hypothesized to be indicative of the habitat affinities of early colonizers [13]. However, this hypothesis has not been tested with ancestral-area reconstruction analyses. As in [6], resolution of deeper nodes in our analysis was inconsistent, weakening any attempts at ancestral reconstructions. Also, uneven geographic sampling in our nuclear dataset indicates that additional populations should be incorporated before carrying out quantitative analysis.

As in the bat genus *Scotophilus* [20], the population-level phylogenetic analyses presented here document repeated patterns of clade replacements between eastern and southern Africa. In the *fumigatus* group, paired clades support replacement between eastern and southern Africa (Fig. 4; *fumigatus/eloquens* 1 + 4 + 8 vs. *fumigatus/eloquens* 6 and *fumigatus/eloquens* 2 + 3 vs. *fumigatus/eloquens* 7). This relationship is also supported by one clade-pair (*clivosus* 1 vs. *clivosus* 3) in the *ferrumequinum* group, and one pair (cf. *landeri* vs. *lobatus*) in the *landeri* group. In the *capensis* group, phylogeographic patterns appear more complex (probably owing to better sampling) and multiple lineages exhibit sympatry. At least one is a putative but undescribed species in sympatry with its sisters in the *capensis* group (cf. *denti/simulator*; Figs. 3 and 4). Sampling is still limited in the rain forests of Central and West Africa, but an enigmatic relationship is apparent in the cyt-*b* tree (Fig. 3), where three newly sequenced specimens from the western Guineo-Congolian rain forest (cf. *denti* in Fig. 3) are sister to arid-land *R. denti*. The concatenated nuclear tree recovers two members of this clade from DRC and Gabon as nested within *R. simulator*. In both datasets an unexpectedly close relationship is inferred for a savanna/woodland habitat species with poorly surveyed populations living in humid rainforest.

Additional insights to Afrotropical *Rhinolophus* are now possible with this greater phylogenetic understanding. The genus is interesting from a public health standpoint owing to various associated viral pathogens [53, 59–61]. Its constant-frequency echolocation calls have been widely studied for their value in communication [62, 63], adaptation and speciation [64–66], and resource subdivision [67, 68]. Their noseleaves, cranial morphology, dentition, and bacula are all richly diversified morphological systems [13, 69] hardly studied from developmental or evolutionary perspectives. Continued efforts to characterize these newly documented lineages across all of these phenotypic dimensions will offer greater understanding of their evolutionary development and diversification.

## Additional files

Additional file 1: List of specimens used in genetic analyses of *Rhinolophus*. (XLSX 55 kb)

Additional file 2: Primer information for regions amplified in the current study. (DOCX 18 kb)

Additional file 3: Uncorrected cyt-*b p*-distances among (below diagonal) and within (numbers on diagonal) African *Rhinolophus* clades calculated in MEGA X 10.0.5 [36]. (XLS 46 kb)

Additional file 4: Maximum likelihood phylogeny of 350 mitochondrial cytochrome-*b* sequences of *Rhinolophus*. The phylogeny was inferred in IQ-TREE and its topology was very similar to the Bayesian phylogeny calculated in MRBAYES. Filled black circles on nodes denote bootstrap values (BS) ≥ 70% and Bayesian posterior probabilities (PP) ≥ 0.95, left-half-filled circles indicate BS ≥ 70% and PP < 0.95, right-half-filled circles indicate BS < 70% and PP ≥ 0.95, and unmarked nodes indicate BS < 70% and PP < 0.95. Branch colors indicate individual clade membership. Species groups are from [13]. Specimen localities include counties for Kenya. Museum acronyms are defined in Additional file 1. (PDF 453 kb)

Additional file 5: Maximum likelihood gene tree inferred for cyt-*b* using IQ-TREE that includes two sequences of *R. gorongosae* deposited in GenBank [12; indicated by red font] and five specimens newly sequenced for cyt-*b* in this study (indicated by blue font). DM14815 is included twice in the tree (both the GenBank sequence and a newly generated sequence from this study). Nodal support is indicated above branches. Museum acronyms are defined in Additional file 1. (PDF 533 kb)

Additional file 6: Maximum likelihood gene trees inferred for nuclear introns using IQ-TREE (A–D) and Bayesian gene trees inferred for nuclear introns using MRBAYES (E–H). Nodal support is indicated above branches. Museum acronyms are defined in Additional file 1. (PDF 1980 kb)

## Data Availability

The DNA sequence data generated for this article are available on GenBank with the following accession numbers: MN025547–MN026153. The DNA sequence alignments used in the analyses for this article have been deposited on Figshare under Accession doi: 10.6084/m9.figshare.8239760).

## Abbreviations

BPP: Bayesian Phylogenetics and Phylogeography
cyt-*b*: Cytochrome-*b*
DM: Durban Natural Science Museum
DRC: Democratic Republic of Congo
ESS: Effective sample size
FMNH: Field Museum of Natural History
ML: Maximum likelihood
Mya: Million years ago
NMK: National Museums of Kenya
PCR: Polymerase chain reaction
PP: Posterior probability
PS: Prior scheme
