# Supplementary material for: Molecular phylogenetics of the African horseshoe bats (Chiroptera: Rhinolophidae): expanded geographic and taxonomic sampling of the Afrotropics
Source: BMC Evol Biol. 2019 Aug 22;19:166. doi: 10.1186/s12862-019-1485-1 (PMC6704657; doi:10.1186/s12862-019-1485-1)
Supplement: Supplementary file 2 — Primer information for regions amplified in the current study. (DOCX 18 kb) [file 12862_2019_1485_MOESM2_ESM.docx]

**Additional file 2**. Primer information for regions amplified in the current study.

| Primer name | Tm | Sequence | Primer publication |
| --- | --- | --- | --- |
| ACOX2-3-F | 61.7 | 5'-CCTSGGCTCDGAGGAGCAGAT-3' | Salicini et al. 2011 |
| ACOX2-3-R | 59.3 | 5'-GGGCTGTGHAYCACAAACTCCT-3' |  |
| COPS7A-4-F | 61.3 | 5'-TACAGCATYGGRCGRGACATCCA-3' | Salicini et al. 2011 |
| COPS7A-4-R | 63.3 | 5'-TCACYTGCTCCTCRATGCCKGACA-3' |  |
| ROGDI-7-F | 62 | 5'-CTGATGGAYGCYGTGATGCTGCA-3' | Salicini et al. 2011 |
| ROGDI-7-R | 61.8 | 5'-CACGGTGAGGCASAGCTTGTTGA-3' |  |
| STAT5b-F | 57.8 | 5'-CTGCTCATCAACAAGCCCGA-3' | Matthee et al. 2001 |
| STAT5b-R | 60.9 | 5'-GGCTTCAGGTTCCACAGGTTGC-3' |  |
| cytb-LGL-765-F | 52.6 | 5'-GAAAAACCAYCGTTGTWATTCAACT-3' | Trujillo et al. 2009 |
| cytb-LGL-766-R | 48.3 | 5'-GTTTAATTAGAATYTYAGCTTTGGG-3' |  |
| cytb-internal-f | 53.6 | 5'-TCATCGCAGCTATAGTAATAGTACA-3' | This study |
| cytb-internal-r | 48.6 | 5'-GGGATTGAGCGTAGAATTGCA-3' |  |

Matthee, C. A., Burzlaff, J. D., Taylor, J. F., Davis, S. K. (2001). Mining the mammalian genome for artiodactyl systematics. *Systematic Biology 50*(3), 367-390.

Salicini, I., Ibáñez, C., Juste, J. (2011). Multilocus phylogeny and species delimitation within the Natterer’s bat species complex in the Western Palearctic. *Molecular Phylogenetics and Evolution,* *61*(3), 888-898.

Trujillo, R. G., Patton, J. C., Schlitter, D. A., and Bickham, J. W. (2009). Molecular phylogenetics of the bat genus *Scotophilus* (Chiroptera: Vespertilionidae): perspectives from paternally and maternally inherited genomes. *Journal of Mammalogy* *90*(3), 548-560.
