# Supplementary material for: Molecular phylogenetics of the African horseshoe bats (Chiroptera: Rhinolophidae): expanded geographic and taxonomic sampling of the Afrotropics
Source: BMC Evol Biol. 2019 Aug 22;19:166. doi: 10.1186/s12862-019-1485-1 (PMC6704657; doi:10.1186/s12862-019-1485-1)

Additional file 4. Maximum likelihood phylogeny of 350 mitochondrial cytochrome-*b* sequences of *Rhinolophus*. The phylogeny was inferred in IQ-TREE and its topology was very similar to the Bayesian phylogeny calculated in MRBAYES. Filled black circles on nodes denote bootstrap values (BS) ≥ 70% and Bayesian posterior probabilities (PP) ≥ 0.95, left-half-filled circles indicate BS ≥ 70% and PP < 0.95, right-half-filled circles indicate BS < 70% and PP ≥ 0.95, and unmarked nodes indicate BS < 70% and PP < 0.95. Branch colors indicate individual clade membership. Species groups are from Csorba et al. (2003). Specimen localities include counties for Kenya. Museum acronyms are defined in Additional file 1.

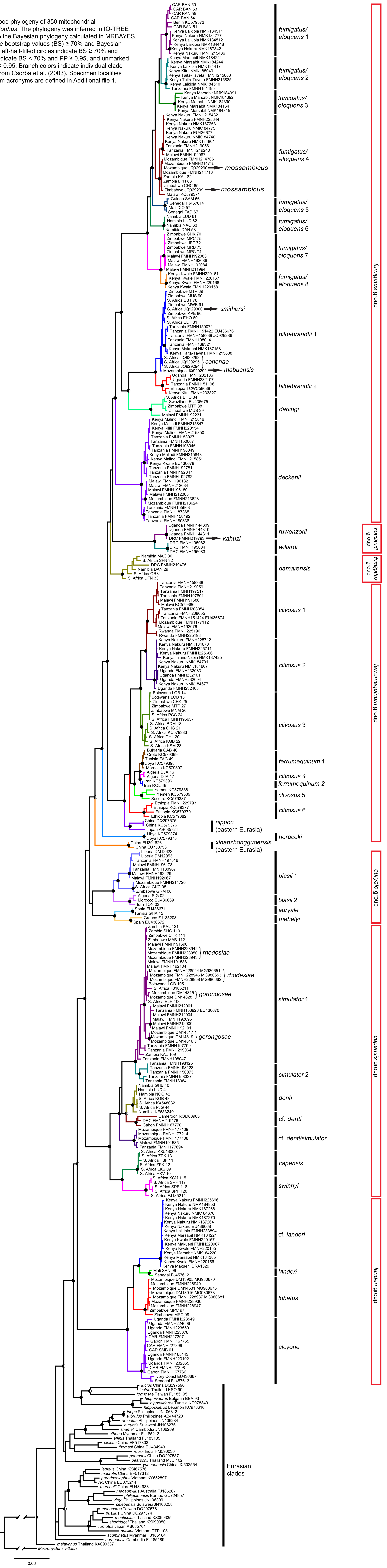

Supplement: Supplementary file 4 — Maximum likelihood phylogeny of 350 mitochondrial cytochrome-b sequences of Rhinolophus. The phylogeny was inferred in IQ-TREE and its topology was very similar to the Bayesian phylogeny calculated in MRBAYES. Filled black circles on nodes denote bootstrap values (BS) ≥ 70% and Bayesian posterior probabilities (PP) ≥ 0.95, left-half-filled circles indicate BS ≥ 70% and PP < 0.95, right-half-filled circles indicate BS < 70% and PP ≥ 0.95, and unmarked nodes indicate BS < 70% and PP < 0.95. Branch colors indicate individual clade membership. Species groups are from [13]. Specimen localities include counties for Kenya. Museum acronyms are defined in Additional file 1. (PDF 453 kb) [file 12862_2019_1485_MOESM4_ESM.pdf]
