# Supplementary material for: Molecular phylogenetics of the African horseshoe bats (Chiroptera: Rhinolophidae): expanded geographic and taxonomic sampling of the Afrotropics
Source: BMC Evol Biol. 2019 Aug 22;19:166. doi: 10.1186/s12862-019-1485-1 (PMC6704657; doi:10.1186/s12862-019-1485-1)

Additional file 6. Maximum likelihood gene trees inferred for nuclear introns using IQ-TREE (**A–D)**) and Bayesian gene trees inferred for nuclear introns using MRBAYES **(E–H).** Boostrap support (**A–D)** and posterior probabilites **(E–H)** are indicated aboves branches. Bootstrap support and posterior probablities < 50% and 0.50, respectively, are not indicated.

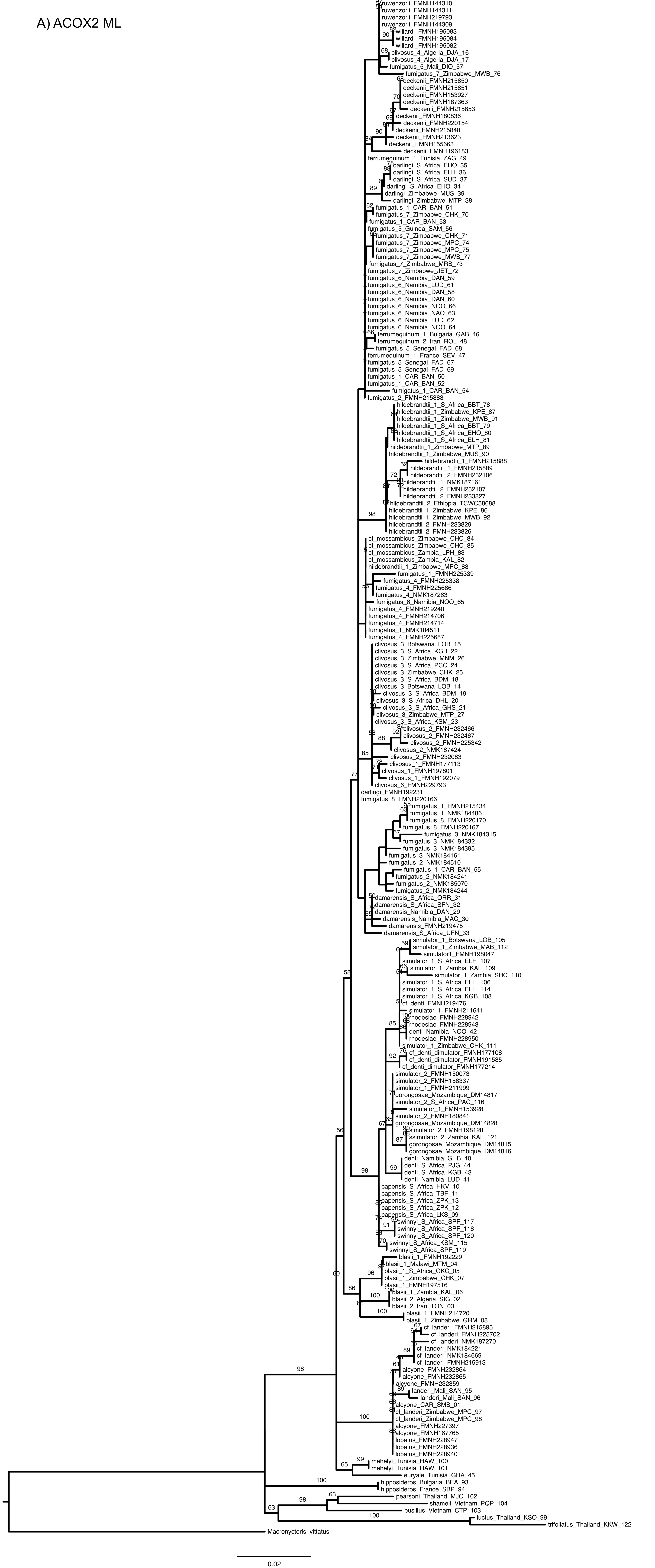

A) ACOX2 ML
ruwenzorii_FMNH144310
ruwenzorii_FMNH144311
ruwenzorii_FMNH219793
ruwenzorii_FMNH144309
willardi_FMNH195083
willardi_FMNH195084
willardi_FMNH195082
clivosus_4_Algeria_DJA_16
clivosus_4_Algeria_DJA_17
fumigatus_5_Mali_DIO_57
fumigatus_7_Zimbabwe_MWB_76
deckenii_FMNH215850
deckenii_FMNH215851
deckenii_FMNH153927
deckenii_FMNH187363
deckenii_FMNH215853
deckenii_FMNH180836
deckenii_FMNH220154
deckenii_FMNH215848
deckenii_FMNH213623
deckenii_FMNH155663
deckenii_FMNH196183
ferrumequinum_1_Tunisia_ZAG_49
darlingi_S_Africa_EHO_35
darlingi_S_Africa_ELH_36
darlingi_S_Africa_SUD_37
darlingi_S_Africa_EHO_34
darlingi_Zimbabwe_MUS_39
darlingi_Zimbabwe_MTP_38
fumigatus_1_CAR_BAN_51
fumigatus_7_Zimbabwe_CHK_70
fumigatus_1_CAR_BAN_53
fumigatus_5_Guinea_SAM_56
fumigatus_7_Zimbabwe_CHK_71
fumigatus_7_Zimbabwe_MPC_74
fumigatus_7_Zimbabwe_MPC_75
fumigatus_7_Zimbabwe_MWB_77
fumigatus_7_Zimbabwe_MRB_73
fumigatus_7_Zimbabwe_JET_72
fumigatus_6_Namibia_DAN_59
fumigatus_6_Namibia_LUD_61
fumigatus_6_Namibia_DAN_58
fumigatus_6_Namibia_DAN_60
fumigatus_6_Namibia_NOO_66
fumigatus_6_Namibia_NAO_63
fumigatus_6_Namibia_LUD_62
fumigatus_6_Namibia_NOO_64
ferrumequinum_1_Bulgaria_GAB_46
ferrumequinum_2_Iran_ROL_48
fumigatus_5_Senegal_FAD_68
ferrumequinum_1_France_SEV_47
fumigatus_5_Senegal_FAD_67
fumigatus_5_Senegal_FAD_69
fumigatus_1_CAR_BAN_50
fumigatus_1_CAR_BAN_52
fumigatus_1_CAR_BAN_54
fumigatus_2_FMNH215883
hildebrandtii_1_S_Africa_BBT_78
hildebrandtii_1_Zimbabwe_KPE_87
hildebrandtii_1_Zimbabwe_MWB_91
hildebrandtii_1_S_Africa_BBT_79
hildebrandtii_1_S_Africa_EHO_80
hildebrandtii_1_S_Africa_ELH_81
hildebrandtii_1_Zimbabwe_MTP_89
hildebrandtii_1_Zimbabwe_MUS_90
hildebrandtii_1_FMNH215888
hildebrandtii_1_FMNH215889
hildebrandtii_2_FMNH232106
hildebrandtii_1_NMK187161
hildebrandtii_2_FMNH232107
hildebrandtii_2_FMNH233827
hildebrandtii_2_Ethiopia_TCWC58688
hildebrandtii_1_Zimbabwe_KPE_86
hildebrandtii_1_Zimbabwe_MWB_92
hildebrandtii_2_FMNH233829
hildebrandtii_2_FMNH233826
cf_mossambicus_Zimbabwe_CHC_84
cf_mossambicus_Zimbabwe_CHC_85
cf_mossambicus_Zambia_LPH_83
cf_mossambicus_Zambia_KAL_82
hildebrandtii_1_Zimbabwe_MPC_88
fumigatus_1_FMNH225339
fumigatus_4_FMNH225338
fumigatus_4_FMNH225686
fumigatus_4_NMK187263
fumigatus_6_Namibia_NOO_65
fumigatus_4_FMNH219240
fumigatus_4_FMNH214706
fumigatus_4_FMNH214714
fumigatus_1_NMK184511
fumigatus_4_FMNH225687
clivosus_3_Botswana_LOB_15
clivosus_3_S_Africa_KGB_22
clivosus_3_Zimbabwe_MNM_26
clivosus_3_S_Africa_PCC_24
clivosus_3_Zimbabwe_CHK_25
clivosus_3_S_Africa_BDM_18
clivosus_3_Botswana_LOB_14
clivosus_3_S_Africa_BDM_19
clivosus_3_S_Africa_DHL_20
clivosus_3_S_Africa_GHS_21
clivosus_3_Zimbabwe_MTP_27
clivosus_3_S_Africa_KSM_23
clivosus_2_FMNH232466
clivosus_2_FMNH232467
clivosus_2_FMNH225342
clivosus_2_NMK187424
clivosus_2_FMNH232083
clivosus_1_FMNH177113
clivosus_1_FMNH197801
clivosus_1_FMNH192079
clivosus_6_FMNH229793
darlingi_FMNH192231
fumigatus_8_FMNH220166
fumigatus_1_FMNH215434
fumigatus_1_NMK184486
fumigatus_8_FMNH220170
fumigatus_8_FMNH220167
fumigatus_3_NMK184315
fumigatus_3_NMK184332
fumigatus_3_NMK184395
fumigatus_3_NMK184161
fumigatus_2_NMK184510
fumigatus_1_CAR_BAN_55
fumigatus_2_NMK184241
fumigatus_2_NMK185070
fumigatus_2_NMK184244
damarensis_S_Africa_ORR_31
damarensis_S_Africa_SFN_32
damarensis_Namibia_DAN_29
damarensis_Namibia_MAC_30
damarensis_FMNH219475
damarensis_S_Africa_UFN_33
simulator_1_Botswana_LOB_105
simulator_1_Zimbabwe_MAB_112
simulator1_FMNH198047
simulator_1_S_Africa_ELH_107
simulator_1_Zambia_KAL_109
simulator_1_Zambia_SHC_110
simulator_1_S_Africa_ELH_106
simulator_1_S_Africa_ELH_114
simulator_1_S_Africa_KGB_108
cf_denti_FMNH219476
simulator_1_FMNH211641
rhodesiae_FMNH228942
rhodesiae_FMNH228943
denti_Namibia_NOO_42
rhodesiae_FMNH228950
simulator_1_Zimbabwe_CHK_111
cf_denti_dimulator_FMNH177108
cf_denti_dimulator_FMNH191585
cf_denti_dimulator_FMNH177214
simulator_2_FMNH150073
simulator_2_FMNH158337
simulator_1_FMNH211999
gorongosae_Mozambique_DM14817
simulator_2_S_Africa_PAC_116
simulator_1_FMNH153928
simulator_2_FMNH180841
gorongosae_Mozambique_DM14828
simulator_2_FMNH198128
ssimulator_2_Zambia_KAL_121
gorongosae_Mozambique_DM14815
gorongosae_Mozambique_DM14816
denti_Namibia_GHB_40
denti_S_Africa_PJG_44
denti_S_Africa_KGB_43
denti_Namibia_LUD_41
capensis_S_Africa_HKV_10
capensis_S_Africa_TBF_11
capensis_S_Africa_ZPK_13
capensis_S_Africa_ZPK_12
capensis_S_Africa_LKS_09
swinnyi_S_Africa_SPF_117
swinnyi_S_Africa_SPF_118
swinnyi_S_Africa_SPF_120
swinnyi_S_Africa_KSM_115
swinnyi_S_Africa_SPF_119
blasii_1_FMNH192229
blasii_1_Malawi_MTM_04
blasii_1_S_Africa_GKC_05
blasii_1_Zimbabwe_CHK_07
blasii_1_FMNH197516
blasii_1_Zambia_KAL_06
blasii_2_Algeria_SIG_02
blasii_2_Iran_TON_03
blasii_1_FMNH214720
blasii_1_Zimbabwe_GRM_08
cf_landeri_FMNH215895
cf_landeri_FMNH225702
cf_landeri_NMK187270
cf_landeri_NMK184221
cf_landeri_NMK184669
cf_landeri_FMNH215913
alcyone_FMNH232864
alcyone_FMNH232865
alcyone_FMNH232859
landeri_Mali_SAN_95
landeri_Mali_SAN_96
alcyone_CAR_SMB_01
cf_landeri_Zimbabwe_MPC_97
cf_landeri_Zimbabwe_MPC_98
alcyone_FMNH227397
alcyone_FMNH167765
lobatus_FMNH228947
lobatus_FMNH228936
lobatus_FMNH228940
mehelyi_Tunisia_HAW_100
mehelyi_Tunisia_HAW_101
euryale_Tunisia_GHA_45
hipposideros_Bulgaria_BEA_93
hipposideros_France_SBP_94
pearsoni_Thailand_MJC_102
shameli_Vietnam_PQP_104
pusillus_Vietnam_CTP_103
luctus_Thailand_KSO_99
trifoliatus_Thailand_KKW_122
Macronycteris_vittatus
0.02

B) COPS7A ML

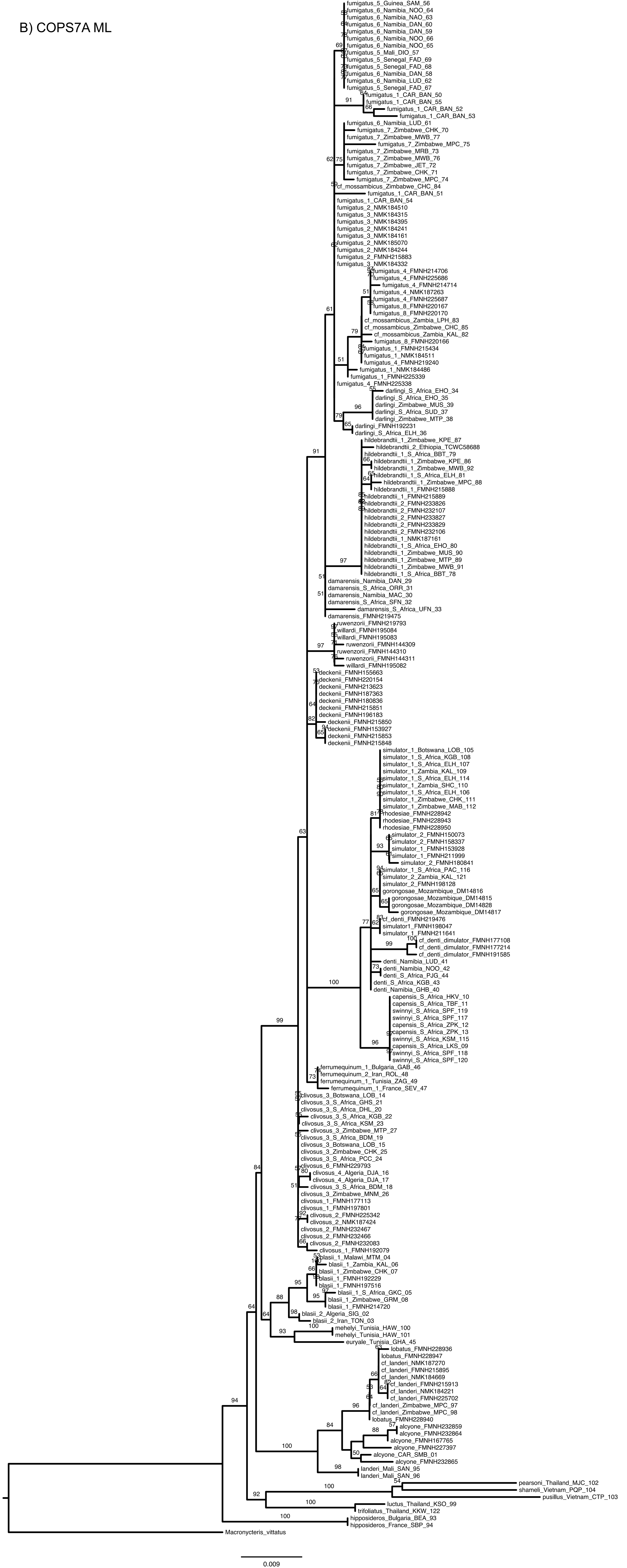

fumigatus_5_Guinea_SAM_56
fumigatus_6_Namibia_NOO_64
fumigatus_6_Namibia_NAO_63
fumigatus_6_Namibia_DAN_60
fumigatus_6_Namibia_DAN_59
fumigatus_6_Namibia_NOO_66
fumigatus_6_Namibia_NOO_65
fumigatus_5_Mali_DIO_57
fumigatus_5_Senegal_FAD_69
fumigatus_5_Senegal_FAD_68
fumigatus_6_Namibia_DAN_58
fumigatus_6_Namibia_LUD_62
fumigatus_5_Senegal_FAD_67
fumigatus_1_CAR_BAN_50
fumigatus_1_CAR_BAN_55
fumigatus_1_CAR_BAN_52
fumigatus_1_CAR_BAN_53
fumigatus_6_Namibia_LUD_61
fumigatus_7_Zimbabwe_CHK_70
fumigatus_7_Zimbabwe_MWB_77
fumigatus_7_Zimbabwe_MPC_75
fumigatus_7_Zimbabwe_MRB_73
fumigatus_7_Zimbabwe_MWB_76
fumigatus_7_Zimbabwe_JET_72
fumigatus_7_Zimbabwe_CHK_71
fumigatus_7_Zimbabwe_MPC_74
cf_mossambicus_Zimbabwe_CHC_84
fumigatus_1_CAR_BAN_51
fumigatus_1_CAR_BAN_54
fumigatus_2_NMK184510
fumigatus_3_NMK184315
fumigatus_3_NMK184395
fumigatus_2_NMK184241
fumigatus_3_NMK184161
fumigatus_2_NMK185070
fumigatus_2_NMK184244
fumigatus_2_FMNH215883
fumigatus_3_NMK184332
fumigatus_4_FMNH214706
fumigatus_4_FMNH225686
fumigatus_4_FMNH214714
fumigatus_4_NMK187263
fumigatus_4_FMNH225687
fumigatus_8_FMNH220167
fumigatus_8_FMNH220170
cf_mossambicus_Zambia_LPH_83
cf_mossambicus_Zimbabwe_CHC_85
cf_mossambicus_Zambia_KAL_82
fumigatus_8_FMNH220166
fumigatus_1_FMNH215434
fumigatus_1_NMK184511
fumigatus_4_FMNH219240
fumigatus_1_NMK184486
fumigatus_1_FMNH225339
fumigatus_4_FMNH225338
darlingi_S_Africa_EHO_34
darlingi_S_Africa_EHO_35
darlingi_Zimbabwe_MUS_39
darlingi_S_Africa_SUD_37
darlingi_Zimbabwe_MTP_38
darlingi_FMNH192231
darlingi_S_Africa_ELH_36
hildebrandtii_1_Zimbabwe_KPE_87
hildebrandtii_2_Ethiopia_TCWC58688
hildebrandtii_1_S_Africa_BBT_79
hildebrandtii_1_Zimbabwe_KPE_86
hildebrandtii_1_Zimbabwe_MWB_92
hildebrandtii_1_S_Africa_ELH_81
hildebrandtii_1_Zimbabwe_MPC_88
hildebrandtii_1_FMNH215888
hildebrandtii_1_FMNH215889
hildebrandtii_2_FMNH233826
hildebrandtii_2_FMNH232107
hildebrandtii_2_FMNH233827
hildebrandtii_2_FMNH233829
hildebrandtii_2_FMNH232106
hildebrandtii_1_NMK187161
hildebrandtii_1_S_Africa_EHO_80
hildebrandtii_1_Zimbabwe_MUS_90
hildebrandtii_1_Zimbabwe_MTP_89
hildebrandtii_1_Zimbabwe_MWB_91
hildebrandtii_1_S_Africa_BBT_78
damarensis_Namibia_DAN_29
damarensis_S_Africa_ORR_31
damarensis_Namibia_MAC_30
damarensis_S_Africa_SFN_32
damarensis_S_Africa_UFN_33
damarensis_FMNH219475
ruwenzorii_FMNH219793
willardi_FMNH195084
willardi_FMNH195083
ruwenzorii_FMNH144309
ruwenzorii_FMNH144310
ruwenzorii_FMNH144311
willardi_FMNH195082
deckenii_FMNH155663
deckenii_FMNH220154
deckenii_FMNH213623
deckenii_FMNH187363
deckenii_FMNH180836
deckenii_FMNH215851
deckenii_FMNH196183
deckenii_FMNH215850
deckenii_FMNH153927
deckenii_FMNH215853
deckenii_FMNH215848
simulator_1_Botswana_LOB_105
simulator_1_S_Africa_KGB_108
simulator_1_S_Africa_ELH_107
simulator_1_Zambia_KAL_109
simulator_1_S_Africa_ELH_114
simulator_1_Zambia_SHC_110
simulator_1_S_Africa_ELH_106
simulator_1_Zimbabwe_CHK_111
simulator_1_Zimbabwe_MAB_112
rhodesiae_FMNH228942
rhodesiae_FMNH228943
rhodesiae_FMNH228950
simulator_2_FMNH150073
simulator_2_FMNH158337
simulator_1_FMNH153928
simulator_1_FMNH211999
simulator_2_FMNH180841
simulator_1_S_Africa_PAC_116
simulator_2_Zambia_KAL_121
simulator_2_FMNH198128
gorongosae_Mozambique_DM14816
gorongosae_Mozambique_DM14815
gorongosae_Mozambique_DM14828
gorongosae_Mozambique_DM14817
cf_denti_FMNH219476
simulator1_FMNH198047
simulator_1_FMNH211641
cf_denti_dimulator_FMNH177108
cf_denti_dimulator_FMNH177214
cf_denti_dimulator_FMNH191585
denti_Namibia_LUD_41
denti_Namibia_NOO_42
denti_S_Africa_PJG_44
denti_S_Africa_KGB_43
denti_Namibia_GHB_40
capensis_S_Africa_HKV_10
capensis_S_Africa_TBF_11
swinnyi_S_Africa_SPF_119
swinnyi_S_Africa_SPF_117
capensis_S_Africa_ZPK_12
capensis_S_Africa_ZPK_13
swinnyi_S_Africa_KSM_115
capensis_S_Africa_LKS_09
swinnyi_S_Africa_SPF_118
swinnyi_S_Africa_SPF_120
ferrumequinum_1_Bulgaria_GAB_46
ferrumequinum_2_Iran_ROL_48
ferrumequinum_1_Tunisia_ZAG_49
ferrumequinum_1_France_SEV_47
clivosus_3_Botswana_LOB_14
clivosus_3_S_Africa_GHS_21
clivosus_3_S_Africa_DHL_20
clivosus_3_S_Africa_KGB_22
clivosus_3_S_Africa_KSM_23
clivosus_3_Zimbabwe_MTP_27
clivosus_3_S_Africa_BDM_19
clivosus_3_Botswana_LOB_15
clivosus_3_Zimbabwe_CHK_25
clivosus_3_S_Africa_PCC_24
clivosus_6_FMNH229793
clivosus_4_Algeria_DJA_16
clivosus_4_Algeria_DJA_17
clivosus_3_S_Africa_BDM_18
clivosus_3_Zimbabwe_MNM_26
clivosus_1_FMNH177113
clivosus_1_FMNH197801
clivosus_2_FMNH225342
clivosus_2_NMK187424
clivosus_2_FMNH232467
clivosus_2_FMNH232466
clivosus_2_FMNH232083
clivosus_1_FMNH192079
blasii_1_Malawi_MTM_04
blasii_1_Zambia_KAL_06
blasii_1_Zimbabwe_CHK_07
blasii_1_FMNH192229
blasii_1_FMNH197516
blasii_1_S_Africa_GKC_05
blasii_1_Zimbabwe_GRM_08
blasii_1_FMNH214720
blasii_2_Algeria_SIG_02
blasii_2_Iran_TON_03
mehelyi_Tunisia_HAW_100
mehelyi_Tunisia_HAW_101
euryale_Tunisia_GHA_45
lobatus_FMNH228936
lobatus_FMNH228947
cf_landeri_NMK187270
cf_landeri_FMNH215895
cf_landeri_NMK184669
cf_landeri_FMNH215913
cf_landeri_NMK184221
cf_landeri_FMNH225702
cf_landeri_Zimbabwe_MPC_97
cf_landeri_Zimbabwe_MPC_98
lobatus_FMNH228940
alcyone_FMNH232859
alcyone_FMNH232864
alcyone_FMNH167765
alcyone_FMNH227397
alcyone_CAR_SMB_01
alcyone_FMNH232865
landeri_Mali_SAN_95
landeri_Mali_SAN_96
pearsoni_Thailand_MJC_102
shameli_Vietnam_PQP_104
pusillus_Vietnam_CTP_103
luctus_Thailand_KSO_99
trifoliatus_Thailand_KKW_122
hipposideros_Bulgaria_BEA_93
hipposideros_France_SBP_94
Macronycteris_vittatus
0.009

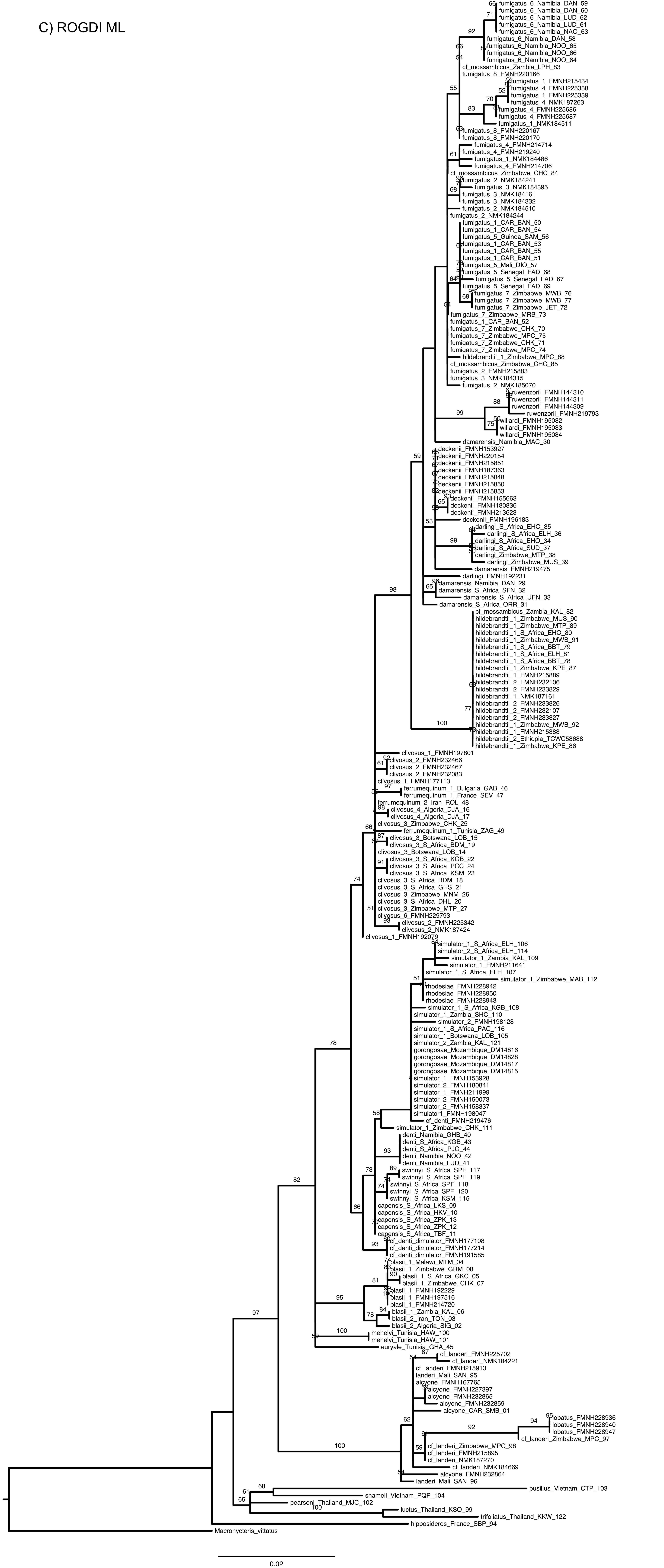
C) ROGDI ML
fumigatus_6_Namibia_DAN_59
fumigatus_6_Namibia_DAN_60
fumigatus_6_Namibia_LUD_62
fumigatus_6_Namibia_LUD_61
fumigatus_6_Namibia_NAO_63
fumigatus_6_Namibia_DAN_58
fumigatus_6_Namibia_NOO_65
fumigatus_6_Namibia_NOO_66
fumigatus_6_Namibia_NOO_64
cf_mossambicus_Zambia_LPH_83
fumigatus_8_FMNH220166
fumigatus_1_FMNH215434
fumigatus_4_FMNH225338
fumigatus_1_FMNH225339
fumigatus_4_NMK187263
fumigatus_4_FMNH225686
fumigatus_4_FMNH225687
fumigatus_1_NMK184511
fumigatus_8_FMNH220167
fumigatus_8_FMNH220170
fumigatus_4_FMNH214714
fumigatus_4_FMNH219240
fumigatus_1_NMK184486
fumigatus_4_FMNH214706
cf_mossambicus_Zimbabwe_CHC_84
fumigatus_2_NMK184241
fumigatus_3_NMK184395
fumigatus_3_NMK184161
fumigatus_3_NMK184332
fumigatus_2_NMK184510
fumigatus_2_NMK184244
fumigatus_1_CAR_BAN_50
fumigatus_1_CAR_BAN_54
fumigatus_5_Guinea_SAM_56
fumigatus_1_CAR_BAN_53
fumigatus_1_CAR_BAN_55
fumigatus_1_CAR_BAN_51
fumigatus_5_Mali_DIO_57
fumigatus_5_Senegal_FAD_68
fumigatus_5_Senegal_FAD_67
fumigatus_5_Senegal_FAD_69
fumigatus_7_Zimbabwe_MWB_76
fumigatus_7_Zimbabwe_MWB_77
fumigatus_7_Zimbabwe_JET_72
fumigatus_7_Zimbabwe_MRB_73
fumigatus_1_CAR_BAN_52
fumigatus_7_Zimbabwe_CHK_70
fumigatus_7_Zimbabwe_MPC_75
fumigatus_7_Zimbabwe_CHK_71
fumigatus_7_Zimbabwe_MPC_74
hildebrandtii_1_Zimbabwe_MPC_88
cf_mossambicus_Zimbabwe_CHC_85
fumigatus_2_FMNH215883
fumigatus_3_NMK184315
fumigatus_2_NMK185070
ruwenzorii_FMNH144310
ruwenzorii_FMNH144311
ruwenzorii_FMNH144309
ruwenzorii_FMNH219793
willardi_FMNH195082
willardi_FMNH195083
willardi_FMNH195084
damarensis_Namibia_MAC_30
deckenii_FMNH153927
deckenii_FMNH220154
deckenii_FMNH215851
deckenii_FMNH187363
deckenii_FMNH215848
deckenii_FMNH215850
deckenii_FMNH215853
deckenii_FMNH155663
deckenii_FMNH180836
deckenii_FMNH213623
deckenii_FMNH196183
darlingi_S_Africa_EHO_35
darlingi_S_Africa_ELH_36
darlingi_S_Africa_EHO_34
darlingi_S_Africa_SUD_37
darlingi_Zimbabwe_MTP_38
darlingi_Zimbabwe_MUS_39
damarensis_FMNH219475
darlingi_FMNH192231
damarensis_Namibia_DAN_29
damarensis_S_Africa_SFN_32
damarensis_S_Africa_UFN_33
damarensis_S_Africa_ORR_31
cf_mossambicus_Zambia_KAL_82
hildebrandtii_1_Zimbabwe_MUS_90
hildebrandtii_1_Zimbabwe_MTP_89
hildebrandtii_1_S_Africa_EHO_80
hildebrandtii_1_Zimbabwe_MWB_91
hildebrandtii_1_S_Africa_BBT_79
hildebrandtii_1_S_Africa_ELH_81
hildebrandtii_1_S_Africa_BBT_78
hildebrandtii_1_Zimbabwe_KPE_87
hildebrandtii_1_FMNH215889
hildebrandtii_2_FMNH232106
hildebrandtii_2_FMNH233829
hildebrandtii_1_NMK187161
hildebrandtii_2_FMNH233826
hildebrandtii_2_FMNH232107
hildebrandtii_2_FMNH233827
hildebrandtii_1_Zimbabwe_MWB_92
hildebrandtii_1_FMNH215888
hildebrandtii_2_Ethiopia_TCWC58688
hildebrandtii_1_Zimbabwe_KPE_86
clivosus_1_FMNH197801
clivosus_2_FMNH232466
clivosus_2_FMNH232467
clivosus_2_FMNH232083
clivosus_1_FMNH177113
ferrumequinum_1_Bulgaria_GAB_46
ferrumequinum_1_France_SEV_47
ferrumequinum_2_Iran_ROL_48
clivosus_4_Algeria_DJA_16
clivosus_4_Algeria_DJA_17
clivosus_3_Zimbabwe_CHK_25
ferrumequinum_1_Tunisia_ZAG_49
clivosus_3_Botswana_LOB_15
clivosus_3_S_Africa_BDM_19
clivosus_3_Botswana_LOB_14
clivosus_3_S_Africa_KGB_22
clivosus_3_S_Africa_PCC_24
clivosus_3_S_Africa_KSM_23
clivosus_3_S_Africa_BDM_18
clivosus_3_S_Africa_GHS_21
clivosus_3_Zimbabwe_MNM_26
clivosus_3_S_Africa_DHL_20
clivosus_3_Zimbabwe_MTP_27
clivosus_6_FMNH229793
clivosus_2_FMNH225342
clivosus_2_NMK187424
clivosus_1_FMNH192079
simulator_1_S_Africa_ELH_106
simulator_2_S_Africa_ELH_114
simulator_1_Zambia_KAL_109
simulator_1_FMNH211641
simulator_1_S_Africa_ELH_107
simulator_1_Zimbabwe_MAB_112
rhodesiae_FMNH228942
rhodesiae_FMNH228950
rhodesiae_FMNH228943
simulator_1_S_Africa_KGB_108
simulator_1_Zambia_SHC_110
simulator_2_FMNH198128
simulator_1_S_Africa_PAC_116
simulator_1_Botswana_LOB_105
simulator_2_Zambia_KAL_121
gorongosae_Mozambique_DM14816
gorongosae_Mozambique_DM14828
gorongosae_Mozambique_DM14817
gorongosae_Mozambique_DM14815
simulator_1_FMNH153928
simulator_2_FMNH180841
simulator_1_FMNH211999
simulator_2_FMNH150073
simulator_2_FMNH158337
simulator1_FMNH198047
cf_denti_FMNH219476
simulator_1_Zimbabwe_CHK_111
denti_Namibia_GHB_40
denti_S_Africa_KGB_43
denti_S_Africa_PJG_44
denti_Namibia_NOO_42
denti_Namibia_LUD_41
swinnyi_S_Africa_SPF_117
swinnyi_S_Africa_SPF_119
swinnyi_S_Africa_SPF_118
swinnyi_S_Africa_SPF_120
swinnyi_S_Africa_KSM_115
capensis_S_Africa_LKS_09
capensis_S_Africa_HKV_10
capensis_S_Africa_ZPK_13
capensis_S_Africa_ZPK_12
capensis_S_Africa_TBF_11
cf_denti_dimulator_FMNH177108
cf_denti_dimulator_FMNH177214
cf_denti_dimulator_FMNH191585
blasii_1_Malawi_MTM_04
blasii_1_Zimbabwe_GRM_08
blasii_1_S_Africa_GKC_05
blasii_1_Zimbabwe_CHK_07
blasii_1_FMNH192229
blasii_1_FMNH197516
blasii_1_FMNH214720
blasii_1_Zambia_KAL_06
blasii_2_Iran_TON_03
blasii_2_Algeria_SIG_02
mehelyi_Tunisia_HAW_100
mehelyi_Tunisia_HAW_101
euryale_Tunisia_GHA_45
cf_landeri_FMNH225702
cf_landeri_NMK184221
cf_landeri_FMNH215913
landeri_Mali_SAN_95
alcyone_FMNH167765
alcyone_FMNH227397
alcyone_FMNH232865
alcyone_FMNH232859
alcyone_CAR_SMB_01
lobatus_FMNH228936
lobatus_FMNH228940
lobatus_FMNH228947
cf_landeri_Zimbabwe_MPC_97
cf_landeri_Zimbabwe_MPC_98
cf_landeri_FMNH215895
cf_landeri_NMK187270
cf_landeri_NMK184669
alcyone_FMNH232864
landeri_Mali_SAN_96
pusillus_Vietnam_CTP_103
shameli_Vietnam_PQP_104
pearsoni_Thailand_MJC_102
luctus_Thailand_KSO_99
trifoliatus_Thailand_KKW_122
hipposideros_France_SBP_94
Macronycteris_vittatus
0.02

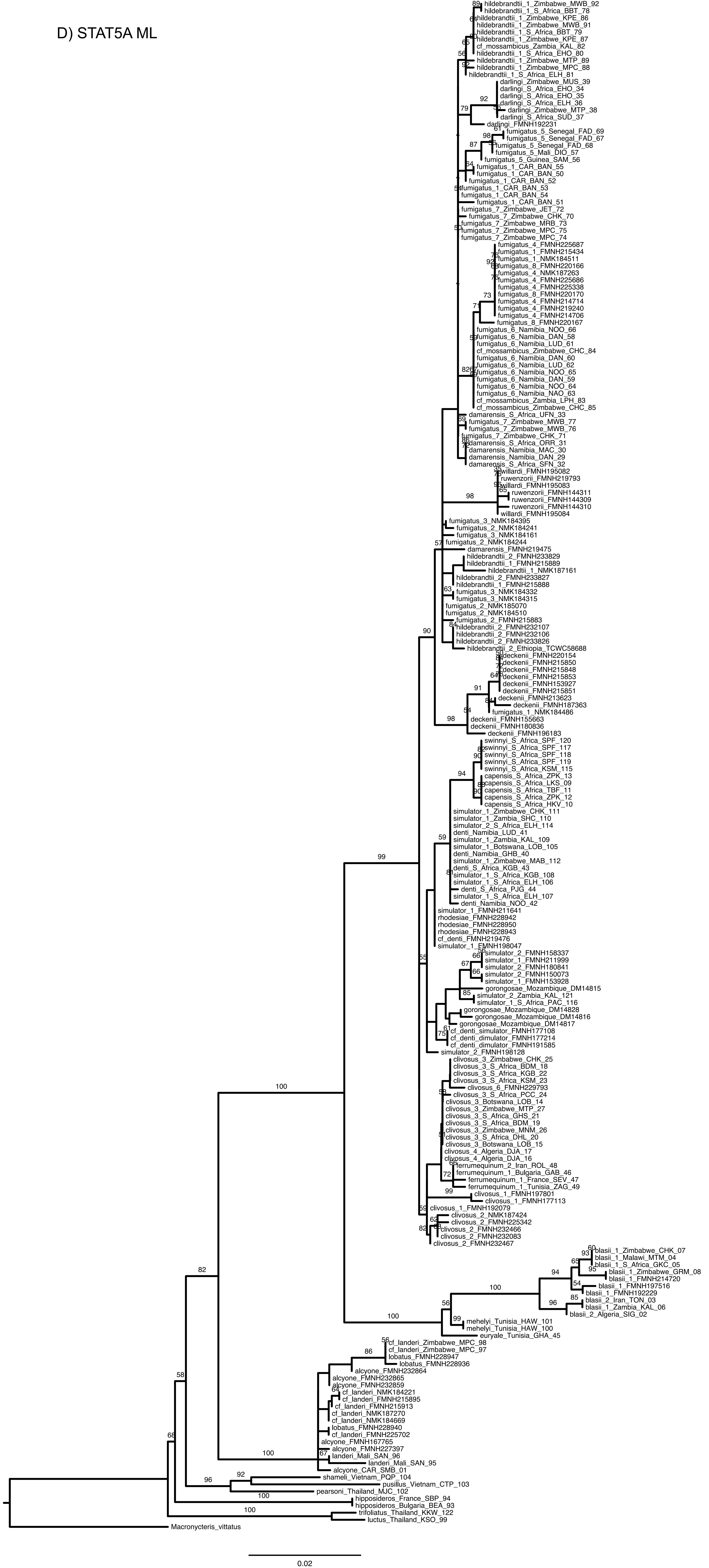
D) STAT5A ML
hildebrandtii_1_Zimbabwe_MWB_92
hildebrandtii_1_S_Africa_BBT_78
hildebrandtii_1_Zimbabwe_KPE_86
hildebrandtii_1_Zimbabwe_MWB_91
hildebrandtii_1_S_Africa_BBT_79
hildebrandtii_1_Zimbabwe_KPE_87
cf_mossambicus_Zambia_KAL_82
hildebrandtii_1_S_Africa_EHO_80
hildebrandtii_1_Zimbabwe_MTP_89
hildebrandtii_1_Zimbabwe_MPC_88
hildebrandtii_1_S_Africa_ELH_81
darlingi_Zimbabwe_MUS_39
darlingi_S_Africa_EHO_34
darlingi_S_Africa_EHO_35
darlingi_S_Africa_ELH_36
darlingi_Zimbabwe_MTP_38
darlingi_S_Africa_SUD_37
darlingi_FMNH192231
fumigatus_5_Senegal_FAD_69
fumigatus_5_Senegal_FAD_67
fumigatus_5_Senegal_FAD_68
fumigatus_5_Mali_DIO_57
fumigatus_5_Guinea_SAM_56
fumigatus_1_CAR_BAN_55
fumigatus_1_CAR_BAN_50
fumigatus_1_CAR_BAN_52
fumigatus_1_CAR_BAN_53
fumigatus_1_CAR_BAN_54
fumigatus_1_CAR_BAN_51
fumigatus_7_Zimbabwe_JET_72
fumigatus_7_Zimbabwe_CHK_70
fumigatus_7_Zimbabwe_MRB_73
fumigatus_7_Zimbabwe_MPC_75
fumigatus_7_Zimbabwe_MPC_74
fumigatus_4_FMNH225687
fumigatus_1_FMNH215434
fumigatus_1_NMK184511
fumigatus_8_FMNH220166
fumigatus_4_NMK187263
fumigatus_4_FMNH225686
fumigatus_4_FMNH225338
fumigatus_8_FMNH220170
fumigatus_4_FMNH214714
fumigatus_4_FMNH219240
fumigatus_4_FMNH214706
fumigatus_8_FMNH220167
fumigatus_6_Namibia_NOO_66
fumigatus_6_Namibia_DAN_58
fumigatus_6_Namibia_LUD_61
cf_mossambicus_Zimbabwe_CHC_84
fumigatus_6_Namibia_DAN_60
fumigatus_6_Namibia_LUD_62
fumigatus_6_Namibia_NOO_65
fumigatus_6_Namibia_DAN_59
fumigatus_6_Namibia_NOO_64
fumigatus_6_Namibia_NAO_63
cf_mossambicus_Zambia_LPH_83
cf_mossambicus_Zimbabwe_CHC_85
damarensis_S_Africa_UFN_33
fumigatus_7_Zimbabwe_MWB_77
fumigatus_7_Zimbabwe_MWB_76
fumigatus_7_Zimbabwe_CHK_71
damarensis_S_Africa_ORR_31
damarensis_Namibia_MAC_30
damarensis_Namibia_DAN_29
damarensis_S_Africa_SFN_32
willardi_FMNH195082
ruwenzorii_FMNH219793
willardi_FMNH195083
ruwenzorii_FMNH144311
ruwenzorii_FMNH144309
ruwenzorii_FMNH144310
willardi_FMNH195084
fumigatus_3_NMK184395
fumigatus_2_NMK184241
fumigatus_3_NMK184161
fumigatus_2_NMK184244
damarensis_FMNH219475
hildebrandtii_2_FMNH233829
hildebrandtii_1_FMNH215889
hildebrandtii_1_NMK187161
hildebrandtii_2_FMNH233827
hildebrandtii_1_FMNH215888
fumigatus_3_NMK184332
fumigatus_3_NMK184315
fumigatus_2_NMK185070
fumigatus_2_NMK184510
fumigatus_2_FMNH215883
hildebrandtii_2_FMNH232107
hildebrandtii_2_FMNH232106
hildebrandtii_2_FMNH233826
hildebrandtii_2_Ethiopia_TCWC58688
deckenii_FMNH220154
deckenii_FMNH215850
deckenii_FMNH215848
deckenii_FMNH215853
deckenii_FMNH153927
deckenii_FMNH215851
deckenii_FMNH213623
deckenii_FMNH187363
fumigatus_1_NMK184486
deckenii_FMNH155663
deckenii_FMNH180836
deckenii_FMNH196183
swinnyi_S_Africa_SPF_120
swinnyi_S_Africa_SPF_117
swinnyi_S_Africa_SPF_118
swinnyi_S_Africa_SPF_119
swinnyi_S_Africa_KSM_115
capensis_S_Africa_ZPK_13
capensis_S_Africa_LKS_09
capensis_S_Africa_TBF_11
capensis_S_Africa_ZPK_12
capensis_S_Africa_HKV_10
simulator_1_Zimbabwe_CHK_111
simulator_1_Zambia_SHC_110
simulator_2_S_Africa_ELH_114
denti_Namibia_LUD_41
simulator_1_Zambia_KAL_109
simulator_1_Botswana_LOB_105
denti_Namibia_GHB_40
simulator_1_Zimbabwe_MAB_112
denti_S_Africa_KGB_43
simulator_1_S_Africa_KGB_108
simulator_1_S_Africa_ELH_106
denti_S_Africa_PJG_44
simulator_1_S_Africa_ELH_107
denti_Namibia_NOO_42
simulator_1_FMNH211641
rhodesiae_FMNH228942
rhodesiae_FMNH228950
rhodesiae_FMNH228943
cf_denti_FMNH219476
simulator_1_FMNH198047
simulator_2_FMNH158337
simulator_1_FMNH211999
simulator_2_FMNH180841
simulator_2_FMNH150073
simulator_1_FMNH153928
gorongosae_Mozambique_DM14815
simulator_2_Zambia_KAL_121
simulator_1_S_Africa_PAC_116
gorongosae_Mozambique_DM14828
gorongosae_Mozambique_DM14816
gorongosae_Mozambique_DM14817
cf_denti_simulator_FMNH177108
cf_denti_dimulator_FMNH177214
cf_denti_dimulator_FMNH191585
simulator_2_FMNH198128
clivosus_3_Zimbabwe_CHK_25
clivosus_3_S_Africa_BDM_18
clivosus_3_S_Africa_KGB_22
clivosus_3_S_Africa_KSM_23
clivosus_6_FMNH229793
clivosus_3_S_Africa_PCC_24
clivosus_3_Botswana_LOB_14
clivosus_3_Zimbabwe_MTP_27
clivosus_3_S_Africa_GHS_21
clivosus_3_S_Africa_BDM_19
clivosus_3_Zimbabwe_MNM_26
clivosus_3_S_Africa_DHL_20
clivosus_3_Botswana_LOB_15
clivosus_4_Algeria_DJA_17
clivosus_4_Algeria_DJA_16
ferrumequinum_2_Iran_ROL_48
ferrumequinum_1_Bulgaria_GAB_46
ferrumequinum_1_France_SEV_47
ferrumequinum_1_Tunisia_ZAG_49
clivosus_1_FMNH197801
clivosus_1_FMNH177113
clivosus_1_FMNH192079
clivosus_2_NMK187424
clivosus_2_FMNH225342
clivosus_2_FMNH232466
clivosus_2_FMNH232083
clivosus_2_FMNH232467
blasii_1_Zimbabwe_CHK_07
blasii_1_Malawi_MTM_04
blasii_1_S_Africa_GKC_05
blasii_1_Zimbabwe_GRM_08
blasii_1_FMNH214720
blasii_1_FMNH197516
blasii_1_FMNH192229
blasii_2_Iran_TON_03
blasii_1_Zambia_KAL_06
blasii_2_Algeria_SIG_02
mehelyi_Tunisia_HAW_101
mehelyi_Tunisia_HAW_100
euryale_Tunisia_GHA_45
cf_landeri_Zimbabwe_MPC_98
cf_landeri_Zimbabwe_MPC_97
lobatus_FMNH228947
lobatus_FMNH228936
alcyone_FMNH232864
alcyone_FMNH232865
alcyone_FMNH232859
cf_landeri_NMK184221
cf_landeri_FMNH215895
cf_landeri_FMNH215913
cf_landeri_NMK187270
cf_landeri_NMK184669
lobatus_FMNH228940
cf_landeri_FMNH225702
alcyone_FMNH167765
alcyone_FMNH227397
landeri_Mali_SAN_96
landeri_Mali_SAN_95
alcyone_CAR_SMB_01
shameli_Vietnam_PQP_104
pusillus_Vietnam_CTP_103
pearsoni_Thailand_MJC_102
hipposideros_France_SBP_94
hipposideros_Bulgaria_BEA_93
trifoliatus_Thailand_KKW_122
luctus_Thailand_KSO_99
Macronycteris_vittatus
0.02

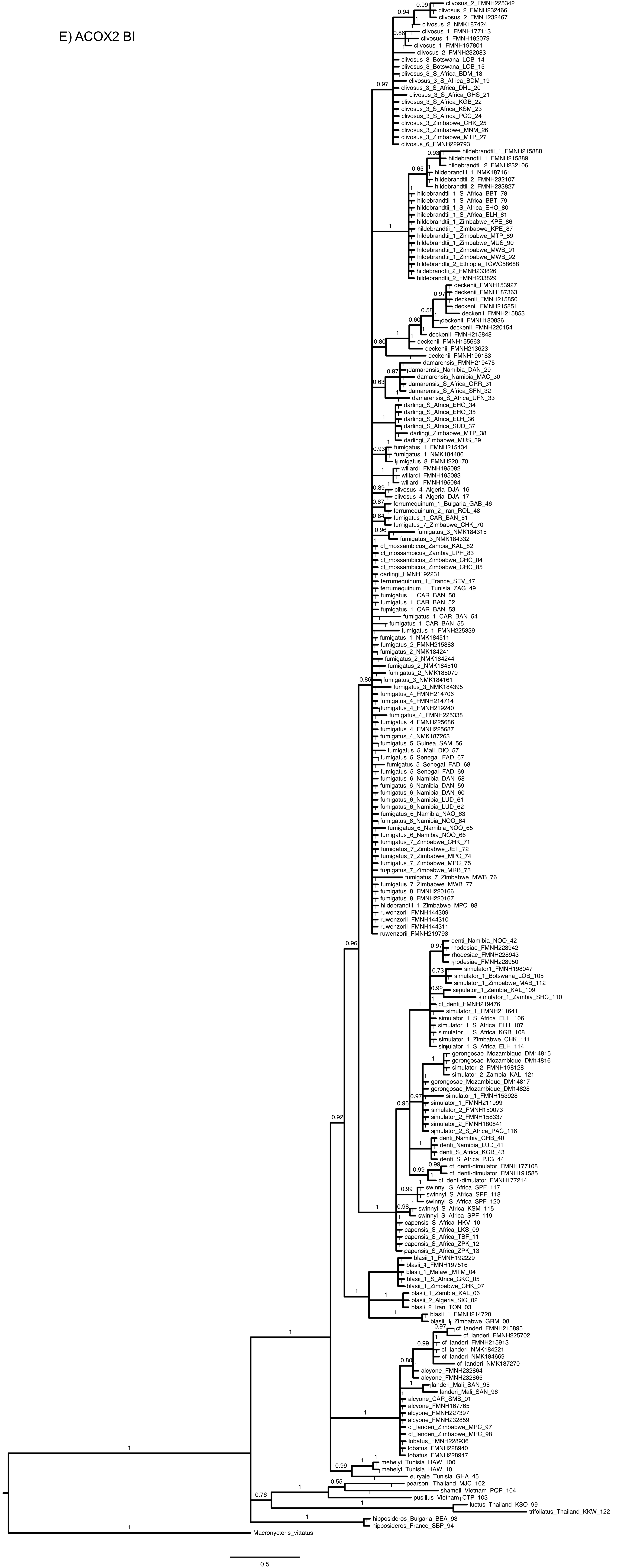
E) ACOX2 BI
clivosus_2_FMNH225342
clivosus_2_FMNH232466
clivosus_2_FMNH232467
clivosus_2_NMK187424
clivosus_1_FMNH177113
clivosus_1_FMNH192079
clivosus_1_FMNH197801
clivosus_2_FMNH232083
clivosus_3_Botswana_LOB_14
clivosus_3_Botswana_LOB_15
clivosus_3_S_Africa_BDM_18
clivosus_3_S_Africa_BDM_19
clivosus_3_S_Africa_DHL_20
clivosus_3_S_Africa_GHS_21
clivosus_3_S_Africa_KGB_22
clivosus_3_S_Africa_KSM_23
clivosus_3_S_Africa_PCC_24
clivosus_3_Zimbabwe_CHK_25
clivosus_3_Zimbabwe_MNM_26
clivosus_3_Zimbabwe_MTP_27
clivosus_6_FMNH229793
hildebrandtii_1_FMNH215888
hildebrandtii_1_FMNH215889
hildebrandtii_2_FMNH232106
hildebrandtii_1_NMK187161
hildebrandtii_2_FMNH232107
hildebrandtii_2_FMNH233827
hildebrandtii_1_S_Africa_BBT_78
hildebrandtii_1_S_Africa_BBT_79
hildebrandtii_1_S_Africa_EHO_80
hildebrandtii_1_S_Africa_ELH_81
hildebrandtii_1_Zimbabwe_KPE_86
hildebrandtii_1_Zimbabwe_KPE_87
hildebrandtii_1_Zimbabwe_MTP_89
hildebrandtii_1_Zimbabwe_MUS_90
hildebrandtii_1_Zimbabwe_MWB_91
hildebrandtii_1_Zimbabwe_MWB_92
hildebrandtii_2_Ethiopia_TCWC58688
hildebrandtii_2_FMNH233826
hildebrandtii_2_FMNH233829
deckenii_FMNH153927
deckenii_FMNH187363
deckenii_FMNH215850
deckenii_FMNH215851
deckenii_FMNH215853
deckenii_FMNH180836
deckenii_FMNH220154
deckenii_FMNH215848
deckenii_FMNH155663
deckenii_FMNH213623
deckenii_FMNH196183
damarensis_FMNH219475
damarensis_Namibia_DAN_29
damarensis_Namibia_MAC_30
damarensis_S_Africa_ORR_31
damarensis_S_Africa_SFN_32
damarensis_S_Africa_UFN_33
darlingi_S_Africa_EHO_34
darlingi_S_Africa_EHO_35
darlingi_S_Africa_ELH_36
darlingi_S_Africa_SUD_37
darlingi_Zimbabwe_MTP_38
darlingi_Zimbabwe_MUS_39
fumigatus_1_FMNH215434
fumigatus_1_NMK184486
fumigatus_8_FMNH220170
willardi_FMNH195082
willardi_FMNH195083
willardi_FMNH195084
clivosus_4_Algeria_DJA_16
clivosus_4_Algeria_DJA_17
ferrumequinum_1_Bulgaria_GAB_46
ferrumequinum_2_Iran_ROL_48
fumigatus_1_CAR_BAN_51
fumigatus_7_Zimbabwe_CHK_70
fumigatus_3_NMK184315
fumigatus_3_NMK184332
cf_mossambicus_Zambia_KAL_82
cf_mossambicus_Zambia_LPH_83
cf_mossambicus_Zimbabwe_CHC_84
cf_mossambicus_Zimbabwe_CHC_85
darlingi_FMNH192231
ferrumequinum_1_France_SEV_47
ferrumequinum_1_Tunisia_ZAG_49
fumigatus_1_CAR_BAN_50
fumigatus_1_CAR_BAN_52
fumigatus_1_CAR_BAN_53
fumigatus_1_CAR_BAN_54
fumigatus_1_CAR_BAN_55
fumigatus_1_FMNH225339
fumigatus_1_NMK184511
fumigatus_2_FMNH215883
fumigatus_2_NMK184241
fumigatus_2_NMK184244
fumigatus_2_NMK184510
fumigatus_2_NMK185070
fumigatus_3_NMK184161
fumigatus_3_NMK184395
fumigatus_4_FMNH214706
fumigatus_4_FMNH214714
fumigatus_4_FMNH219240
fumigatus_4_FMNH225338
fumigatus_4_FMNH225686
fumigatus_4_FMNH225687
fumigatus_4_NMK187263
fumigatus_5_Guinea_SAM_56
fumigatus_5_Mali_DIO_57
fumigatus_5_Senegal_FAD_67
fumigatus_5_Senegal_FAD_68
fumigatus_5_Senegal_FAD_69
fumigatus_6_Namibia_DAN_58
fumigatus_6_Namibia_DAN_59
fumigatus_6_Namibia_DAN_60
fumigatus_6_Namibia_LUD_61
fumigatus_6_Namibia_LUD_62
fumigatus_6_Namibia_NAO_63
fumigatus_6_Namibia_NOO_64
fumigatus_6_Namibia_NOO_65
fumigatus_6_Namibia_NOO_66
fumigatus_7_Zimbabwe_CHK_71
fumigatus_7_Zimbabwe_JET_72
fumigatus_7_Zimbabwe_MPC_74
fumigatus_7_Zimbabwe_MPC_75
fumigatus_7_Zimbabwe_MRB_73
fumigatus_7_Zimbabwe_MWB_76
fumigatus_7_Zimbabwe_MWB_77
fumigatus_8_FMNH220166
fumigatus_8_FMNH220167
hildebrandtii_1_Zimbabwe_MPC_88
ruwenzorii_FMNH144309
ruwenzorii_FMNH144310
ruwenzorii_FMNH144311
ruwenzorii_FMNH219793
denti_Namibia_NOO_42
rhodesiae_FMNH228942
rhodesiae_FMNH228943
rhodesiae_FMNH228950
simulator1_FMNH198047
simulator_1_Botswana_LOB_105
simulator_1_Zimbabwe_MAB_112
simulator_1_Zambia_KAL_109
simulator_1_Zambia_SHC_110
cf_denti_FMNH219476
simulator_1_FMNH211641
simulator_1_S_Africa_ELH_106
simulator_1_S_Africa_ELH_107
simulator_1_S_Africa_KGB_108
simulator_1_Zimbabwe_CHK_111
simulator_1_S_Africa_ELH_114
gorongosae_Mozambique_DM14815
gorongosae_Mozambique_DM14816
simulator_2_FMNH198128
simulator_2_Zambia_KAL_121
gorongosae_Mozambique_DM14817
gorongosae_Mozambique_DM14828
simulator_1_FMNH153928
simulator_1_FMNH211999
simulator_2_FMNH150073
simulator_2_FMNH158337
simulator_2_FMNH180841
simulator_2_S_Africa_PAC_116
denti_Namibia_GHB_40
denti_Namibia_LUD_41
denti_S_Africa_KGB_43
denti_S_Africa_PJG_44
cf_denti-dimulator_FMNH177108
cf_denti-dimulator_FMNH191585
cf_denti-dimulator_FMNH177214
swinnyi_S_Africa_SPF_117
swinnyi_S_Africa_SPF_118
swinnyi_S_Africa_SPF_120
swinnyi_S_Africa_KSM_115
swinnyi_S_Africa_SPF_119
capensis_S_Africa_HKV_10
capensis_S_Africa_LKS_09
capensis_S_Africa_TBF_11
capensis_S_Africa_ZPK_12
capensis_S_Africa_ZPK_13
blasii_1_FMNH192229
blasii_1_FMNH197516
blasii_1_Malawi_MTM_04
blasii_1_S_Africa_GKC_05
blasii_1_Zimbabwe_CHK_07
blasii_1_Zambia_KAL_06
blasii_2_Algeria_SIG_02
blasii_2_Iran_TON_03
blasii_1_FMNH214720
blasii_1_Zimbabwe_GRM_08
cf_landeri_FMNH215895
cf_landeri_FMNH225702
cf_landeri_FMNH215913
cf_landeri_NMK184221
cf_landeri_NMK184669
cf_landeri_NMK187270
alcyone_FMNH232864
alcyone_FMNH232865
landeri_Mali_SAN_95
landeri_Mali_SAN_96
alcyone_CAR_SMB_01
alcyone_FMNH167765
alcyone_FMNH227397
alcyone_FMNH232859
cf_landeri_Zimbabwe_MPC_97
cf_landeri_Zimbabwe_MPC_98
lobatus_FMNH228936
lobatus_FMNH228940
lobatus_FMNH228947
mehelyi_Tunisia_HAW_100
mehelyi_Tunisia_HAW_101
euryale_Tunisia_GHA_45
pearsoni_Thailand_MJC_102
shameli_Vietnam_PQP_104
pusillus_Vietnam_CTP_103
luctus_Thailand_KSO_99
trifoliatus_Thailand_KKW_122
hipposideros_Bulgaria_BEA_93
hipposideros_France_SBP_94
Macronycteris_vittatus
0.5

F) COPS7A BI

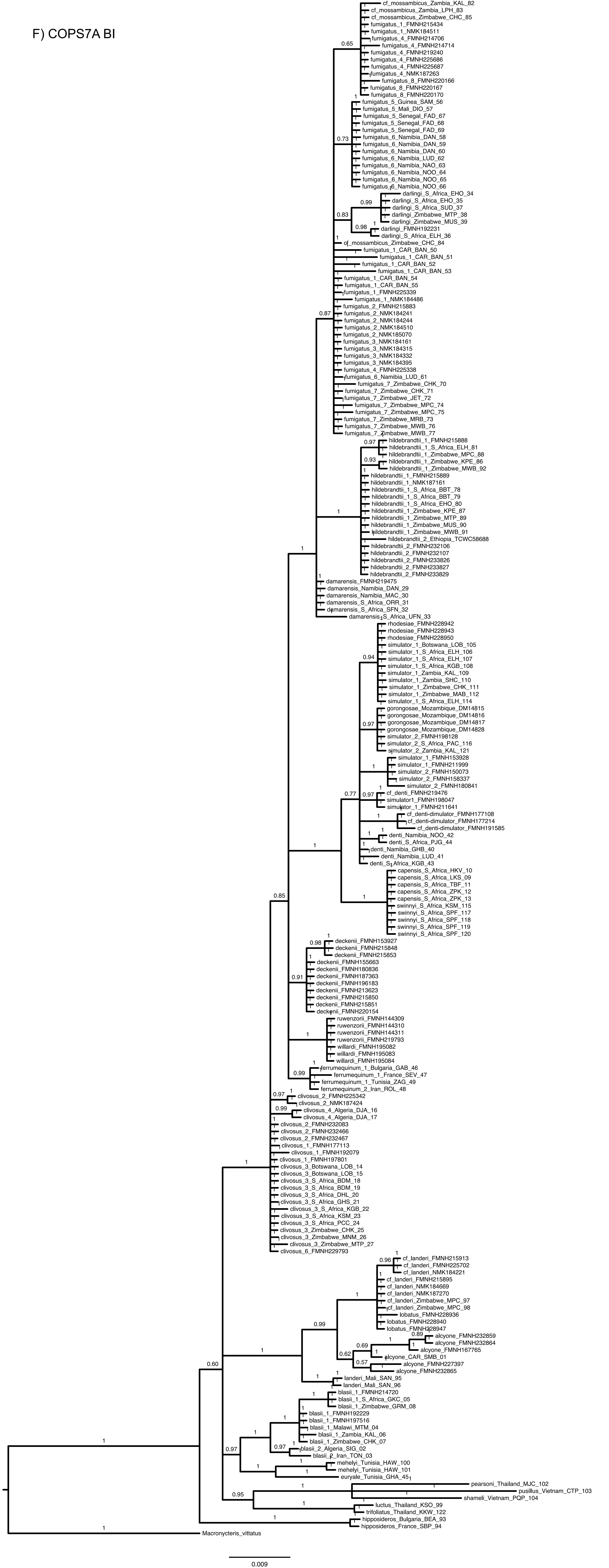
cf_mossambicus_Zambia_KAL_82
cf_mossambicus_Zambia_LPH_83
cf_mossambicus_Zimbabwe_CHC_85
fumigatus_1_FMNH215434
fumigatus_1_NMK184511
fumigatus_4_FMNH214706
fumigatus_4_FMNH214714
fumigatus_4_FMNH219240
fumigatus_4_FMNH225686
fumigatus_4_FMNH225687
fumigatus_4_NMK187263
fumigatus_8_FMNH220166
fumigatus_8_FMNH220167
fumigatus_8_FMNH220170
fumigatus_5_Guinea_SAM_56
fumigatus_5_Mali_DIO_57
fumigatus_5_Senegal_FAD_67
fumigatus_5_Senegal_FAD_68
fumigatus_5_Senegal_FAD_69
fumigatus_6_Namibia_DAN_58
fumigatus_6_Namibia_DAN_59
fumigatus_6_Namibia_DAN_60
fumigatus_6_Namibia_LUD_62
fumigatus_6_Namibia_NAO_63
fumigatus_6_Namibia_NOO_64
fumigatus_6_Namibia_NOO_65
fumigatus_6_Namibia_NOO_66
darlingi_S_Africa_EHO_34
darlingi_S_Africa_EHO_35
darlingi_S_Africa_SUD_37
darlingi_Zimbabwe_MTP_38
darlingi_Zimbabwe_MUS_39
darlingi_FMNH192231
darlingi_S_Africa_ELH_36
cf_mossambicus_Zimbabwe_CHC_84
fumigatus_1_CAR_BAN_50
fumigatus_1_CAR_BAN_51
fumigatus_1_CAR_BAN_52
fumigatus_1_CAR_BAN_53
fumigatus_1_CAR_BAN_54
fumigatus_1_CAR_BAN_55
fumigatus_1_FMNH225339
fumigatus_1_NMK184486
fumigatus_2_FMNH215883
fumigatus_2_NMK184241
fumigatus_2_NMK184244
fumigatus_2_NMK184510
fumigatus_2_NMK185070
fumigatus_3_NMK184161
fumigatus_3_NMK184315
fumigatus_3_NMK184332
fumigatus_3_NMK184395
fumigatus_4_FMNH225338
fumigatus_6_Namibia_LUD_61
fumigatus_7_Zimbabwe_CHK_70
fumigatus_7_Zimbabwe_CHK_71
fumigatus_7_Zimbabwe_JET_72
fumigatus_7_Zimbabwe_MPC_74
fumigatus_7_Zimbabwe_MPC_75
fumigatus_7_Zimbabwe_MRB_73
fumigatus_7_Zimbabwe_MWB_76
fumigatus_7_Zimbabwe_MWB_77
hildebrandtii_1_FMNH215888
hildebrandtii_1_S_Africa_ELH_81
hildebrandtii_1_Zimbabwe_MPC_88
hildebrandtii_1_Zimbabwe_KPE_86
hildebrandtii_1_Zimbabwe_MWB_92
hildebrandtii_1_FMNH215889
hildebrandtii_1_NMK187161
hildebrandtii_1_S_Africa_BBT_78
hildebrandtii_1_S_Africa_BBT_79
hildebrandtii_1_S_Africa_EHO_80
hildebrandtii_1_Zimbabwe_KPE_87
hildebrandtii_1_Zimbabwe_MTP_89
hildebrandtii_1_Zimbabwe_MUS_90
hildebrandtii_1_Zimbabwe_MWB_91
hildebrandtii_2_Ethiopia_TCWC58688
hildebrandtii_2_FMNH232106
hildebrandtii_2_FMNH232107
hildebrandtii_2_FMNH233826
hildebrandtii_2_FMNH233827
hildebrandtii_2_FMNH233829
damarensis_FMNH219475
damarensis_Namibia_DAN_29
damarensis_Namibia_MAC_30
damarensis_S_Africa_ORR_31
damarensis_S_Africa_SFN_32
damarensis_S_Africa_UFN_33
rhodesiae_FMNH228942
rhodesiae_FMNH228943
rhodesiae_FMNH228950
simulator_1_Botswana_LOB_105
simulator_1_S_Africa_ELH_106
simulator_1_S_Africa_ELH_107
simulator_1_S_Africa_KGB_108
simulator_1_Zambia_KAL_109
simulator_1_Zambia_SHC_110
simulator_1_Zimbabwe_CHK_111
simulator_1_Zimbabwe_MAB_112
simulator_1_S_Africa_ELH_114
gorongosae_Mozambique_DM14815
gorongosae_Mozambique_DM14816
gorongosae_Mozambique_DM14817
gorongosae_Mozambique_DM14828
simulator_2_FMNH198128
simulator_2_S_Africa_PAC_116
simulator_2_Zambia_KAL_121
simulator_1_FMNH153928
simulator_1_FMNH211999
simulator_2_FMNH150073
simulator_2_FMNH158337
simulator_2_FMNH180841
cf_denti_FMNH219476
simulator1_FMNH198047
simulator_1_FMNH211641
cf_denti-dimulator_FMNH177108
cf_denti-dimulator_FMNH177214
cf_denti-dimulator_FMNH191585
denti_Namibia_NOO_42
denti_S_Africa_PJG_44
denti_Namibia_GHB_40
denti_Namibia_LUD_41
denti_S_Africa_KGB_43
capensis_S_Africa_HKV_10
capensis_S_Africa_LKS_09
capensis_S_Africa_TBF_11
capensis_S_Africa_ZPK_12
capensis_S_Africa_ZPK_13
swinnyi_S_Africa_KSM_115
swinnyi_S_Africa_SPF_117
swinnyi_S_Africa_SPF_118
swinnyi_S_Africa_SPF_119
swinnyi_S_Africa_SPF_120
deckenii_FMNH153927
deckenii_FMNH215848
deckenii_FMNH215853
deckenii_FMNH155663
deckenii_FMNH180836
deckenii_FMNH187363
deckenii_FMNH196183
deckenii_FMNH213623
deckenii_FMNH215850
deckenii_FMNH215851
deckenii_FMNH220154
ruwenzorii_FMNH144309
ruwenzorii_FMNH144310
ruwenzorii_FMNH144311
ruwenzorii_FMNH219793
willardi_FMNH195082
willardi_FMNH195083
willardi_FMNH195084
ferrumequinum_1_Bulgaria_GAB_46
ferrumequinum_1_France_SEV_47
ferrumequinum_1_Tunisia_ZAG_49
ferrumequinum_2_Iran_ROL_48
clivosus_2_FMNH225342
clivosus_2_NMK187424
clivosus_4_Algeria_DJA_16
clivosus_4_Algeria_DJA_17
clivosus_2_FMNH232083
clivosus_2_FMNH232466
clivosus_2_FMNH232467
clivosus_1_FMNH177113
clivosus_1_FMNH192079
clivosus_1_FMNH197801
clivosus_3_Botswana_LOB_14
clivosus_3_Botswana_LOB_15
clivosus_3_S_Africa_BDM_18
clivosus_3_S_Africa_BDM_19
clivosus_3_S_Africa_DHL_20
clivosus_3_S_Africa_GHS_21
clivosus_3_S_Africa_KGB_22
clivosus_3_S_Africa_KSM_23
clivosus_3_S_Africa_PCC_24
clivosus_3_Zimbabwe_CHK_25
clivosus_3_Zimbabwe_MNM_26
clivosus_3_Zimbabwe_MTP_27
clivosus_6_FMNH229793
cf_landeri_FMNH215913
cf_landeri_FMNH225702
cf_landeri_NMK184221
cf_landeri_FMNH215895
cf_landeri_NMK184669
cf_landeri_NMK187270
cf_landeri_Zimbabwe_MPC_97
cf_landeri_Zimbabwe_MPC_98
lobatus_FMNH228936
lobatus_FMNH228940
lobatus_FMNH228947
alcyone_FMNH232859
alcyone_FMNH232864
alcyone_FMNH167765
alcyone_CAR_SMB_01
alcyone_FMNH227397
alcyone_FMNH232865
landeri_Mali_SAN_95
landeri_Mali_SAN_96
blasii_1_FMNH214720
blasii_1_S_Africa_GKC_05
blasii_1_Zimbabwe_GRM_08
blasii_1_FMNH192229
blasii_1_FMNH197516
blasii_1_Malawi_MTM_04
blasii_1_Zambia_KAL_06
blasii_1_Zimbabwe_CHK_07
blasii_2_Algeria_SIG_02
blasii_2_Iran_TON_03
mehelyi_Tunisia_HAW_100
mehelyi_Tunisia_HAW_101
euryale_Tunisia_GHA_45
pearsoni_Thailand_MJC_102
pusillus_Vietnam_CTP_103
shameli_Vietnam_PQP_104
luctus_Thailand_KSO_99
trifoliatus_Thailand_KKW_122
hipposideros_Bulgaria_BEA_93
hipposideros_France_SBP_94
Macronycteris_vittatus
0.009

G) ROGDI BI

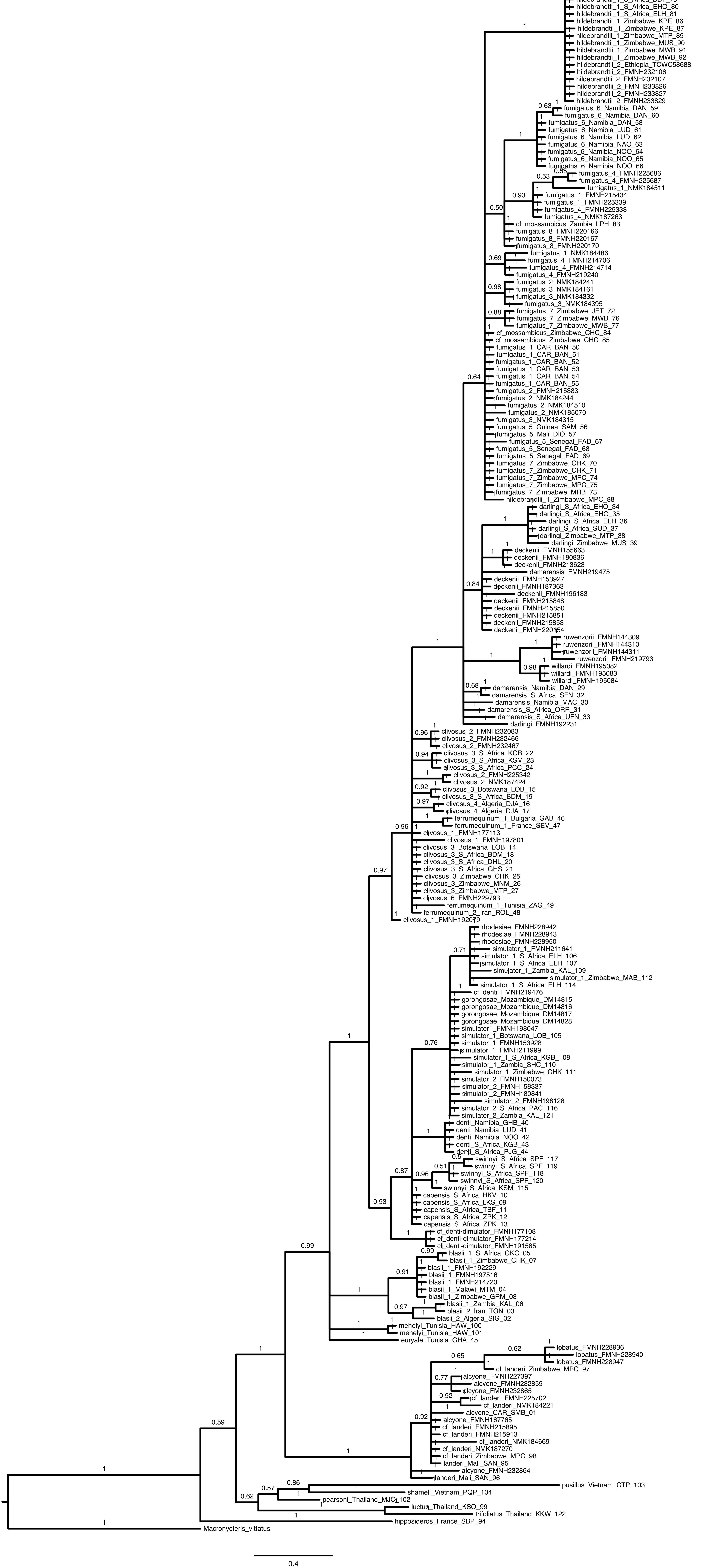
cf_mossambicus_Zambia_KAL_82
hildebrandtii_1_FMNH215888
hildebrandtii_1_FMNH215889
hildebrandtii_1_NMK187161
hildebrandtii_1_S_Africa_BBT_78
hildebrandtii_1_S_Africa_BBT_79
hildebrandtii_1_S_Africa_EHO_80
hildebrandtii_1_S_Africa_ELH_81
hildebrandtii_1_Zimbabwe_KPE_86
hildebrandtii_1_Zimbabwe_KPE_87
hildebrandtii_1_Zimbabwe_MTP_89
hildebrandtii_1_Zimbabwe_MUS_90
hildebrandtii_1_Zimbabwe_MWB_91
hildebrandtii_1_Zimbabwe_MWB_92
hildebrandtii_2_Ethiopia_TCWC58688
hildebrandtii_2_FMNH232106
hildebrandtii_2_FMNH232107
hildebrandtii_2_FMNH233826
hildebrandtii_2_FMNH233827
hildebrandtii_2_FMNH233829
fumigatus_6_Namibia_DAN_59
fumigatus_6_Namibia_DAN_60
fumigatus_6_Namibia_DAN_58
fumigatus_6_Namibia_LUD_61
fumigatus_6_Namibia_LUD_62
fumigatus_6_Namibia_NAO_63
fumigatus_6_Namibia_NOO_64
fumigatus_6_Namibia_NOO_65
fumigatus_6_Namibia_NOO_66
fumigatus_4_FMNH225686
fumigatus_4_FMNH225687
fumigatus_1_NMK184511
fumigatus_1_FMNH215434
fumigatus_1_FMNH225339
fumigatus_4_FMNH225338
fumigatus_4_NMK187263
cf_mossambicus_Zambia_LPH_83
fumigatus_8_FMNH220166
fumigatus_8_FMNH220167
fumigatus_8_FMNH220170
fumigatus_1_NMK184486
fumigatus_4_FMNH214706
fumigatus_4_FMNH214714
fumigatus_4_FMNH219240
fumigatus_2_NMK184241
fumigatus_3_NMK184161
fumigatus_3_NMK184332
fumigatus_3_NMK184395
fumigatus_7_Zimbabwe_JET_72
fumigatus_7_Zimbabwe_MWB_76
fumigatus_7_Zimbabwe_MWB_77
cf_mossambicus_Zimbabwe_CHC_84
cf_mossambicus_Zimbabwe_CHC_85
fumigatus_1_CAR_BAN_50
fumigatus_1_CAR_BAN_51
fumigatus_1_CAR_BAN_52
fumigatus_1_CAR_BAN_53
fumigatus_1_CAR_BAN_54
fumigatus_1_CAR_BAN_55
fumigatus_2_FMNH215883
fumigatus_2_NMK184244
fumigatus_2_NMK184510
fumigatus_2_NMK185070
fumigatus_3_NMK184315
fumigatus_5_Guinea_SAM_56
fumigatus_5_Mali_DIO_57
fumigatus_5_Senegal_FAD_67
fumigatus_5_Senegal_FAD_68
fumigatus_5_Senegal_FAD_69
fumigatus_7_Zimbabwe_CHK_70
fumigatus_7_Zimbabwe_CHK_71
fumigatus_7_Zimbabwe_MPC_74
fumigatus_7_Zimbabwe_MPC_75
fumigatus_7_Zimbabwe_MRB_73
hildebrandtii_1_Zimbabwe_MPC_88
darlingi_S_Africa_EHO_34
darlingi_S_Africa_EHO_35
darlingi_S_Africa_ELH_36
darlingi_S_Africa_SUD_37
darlingi_Zimbabwe_MTP_38
darlingi_Zimbabwe_MUS_39
deckenii_FMNH155663
deckenii_FMNH180836
deckenii_FMNH213623
damarensis_FMNH219475
deckenii_FMNH153927
deckenii_FMNH187363
deckenii_FMNH196183
deckenii_FMNH215848
deckenii_FMNH215850
deckenii_FMNH215851
deckenii_FMNH215853
deckenii_FMNH220154
ruwenzorii_FMNH144309
ruwenzorii_FMNH144310
ruwenzorii_FMNH144311
ruwenzorii_FMNH219793
willardi_FMNH195082
willardi_FMNH195083
willardi_FMNH195084
damarensis_Namibia_DAN_29
damarensis_S_Africa_SFN_32
damarensis_Namibia_MAC_30
damarensis_S_Africa_ORR_31
damarensis_S_Africa_UFN_33
darlingi_FMNH192231
clivosus_2_FMNH232083
clivosus_2_FMNH232466
clivosus_2_FMNH232467
clivosus_3_S_Africa_KGB_22
clivosus_3_S_Africa_KSM_23
clivosus_3_S_Africa_PCC_24
clivosus_2_FMNH225342
clivosus_2_NMK187424
clivosus_3_Botswana_LOB_15
clivosus_3_S_Africa_BDM_19
clivosus_4_Algeria_DJA_16
clivosus_4_Algeria_DJA_17
ferrumequinum_1_Bulgaria_GAB_46
ferrumequinum_1_France_SEV_47
clivosus_1_FMNH177113
clivosus_1_FMNH197801
clivosus_3_Botswana_LOB_14
clivosus_3_S_Africa_BDM_18
clivosus_3_S_Africa_DHL_20
clivosus_3_S_Africa_GHS_21
clivosus_3_Zimbabwe_CHK_25
clivosus_3_Zimbabwe_MNM_26
clivosus_3_Zimbabwe_MTP_27
clivosus_6_FMNH229793
ferrumequinum_1_Tunisia_ZAG_49
ferrumequinum_2_Iran_ROL_48
clivosus_1_FMNH192079
rhodesiae_FMNH228942
rhodesiae_FMNH228943
rhodesiae_FMNH228950
simulator_1_FMNH211641
simulator_1_S_Africa_ELH_106
simulator_1_S_Africa_ELH_107
simulator_1_Zambia_KAL_109
simulator_1_Zimbabwe_MAB_112
simulator_1_S_Africa_ELH_114
cf_denti_FMNH219476
gorongosae_Mozambique_DM14815
gorongosae_Mozambique_DM14816
gorongosae_Mozambique_DM14817
gorongosae_Mozambique_DM14828
simulator1_FMNH198047
simulator_1_Botswana_LOB_105
simulator_1_FMNH153928
simulator_1_FMNH211999
simulator_1_S_Africa_KGB_108
simulator_1_Zambia_SHC_110
simulator_1_Zimbabwe_CHK_111
simulator_2_FMNH150073
simulator_2_FMNH158337
simulator_2_FMNH180841
simulator_2_FMNH198128
simulator_2_S_Africa_PAC_116
simulator_2_Zambia_KAL_121
denti_Namibia_GHB_40
denti_Namibia_LUD_41
denti_Namibia_NOO_42
denti_S_Africa_KGB_43
denti_S_Africa_PJG_44
swinnyi_S_Africa_SPF_117
swinnyi_S_Africa_SPF_119
swinnyi_S_Africa_SPF_118
swinnyi_S_Africa_SPF_120
swinnyi_S_Africa_KSM_115
capensis_S_Africa_HKV_10
capensis_S_Africa_LKS_09
capensis_S_Africa_TBF_11
capensis_S_Africa_ZPK_12
capensis_S_Africa_ZPK_13
cf_denti-dimulator_FMNH177108
cf_denti-dimulator_FMNH177214
cf_denti-dimulator_FMNH191585
blasii_1_S_Africa_GKC_05
blasii_1_Zimbabwe_CHK_07
blasii_1_FMNH192229
blasii_1_FMNH197516
blasii_1_FMNH214720
blasii_1_Malawi_MTM_04
blasii_1_Zimbabwe_GRM_08
blasii_1_Zambia_KAL_06
blasii_2_Iran_TON_03
blasii_2_Algeria_SIG_02
mehelyi_Tunisia_HAW_100
mehelyi_Tunisia_HAW_101
euryale_Tunisia_GHA_45
lobatus_FMNH228936
lobatus_FMNH228940
lobatus_FMNH228947
cf_landeri_Zimbabwe_MPC_97
alcyone_FMNH227397
alcyone_FMNH232859
alcyone_FMNH232865
cf_landeri_FMNH225702
cf_landeri_NMK184221
alcyone_CAR_SMB_01
alcyone_FMNH167765
cf_landeri_FMNH215895
cf_landeri_FMNH215913
cf_landeri_NMK184669
cf_landeri_NMK187270
cf_landeri_Zimbabwe_MPC_98
landeri_Mali_SAN_95
alcyone_FMNH232864
landeri_Mali_SAN_96
pusillus_Vietnam_CTP_103
shameli_Vietnam_PQP_104
pearsoni_Thailand_MJC_102
luctus_Thailand_KSO_99
trifoliatus_Thailand_KKW_122
hipposideros_France_SBP_94
Macronycteris_vittatus
0.4

H) STAT5A BI

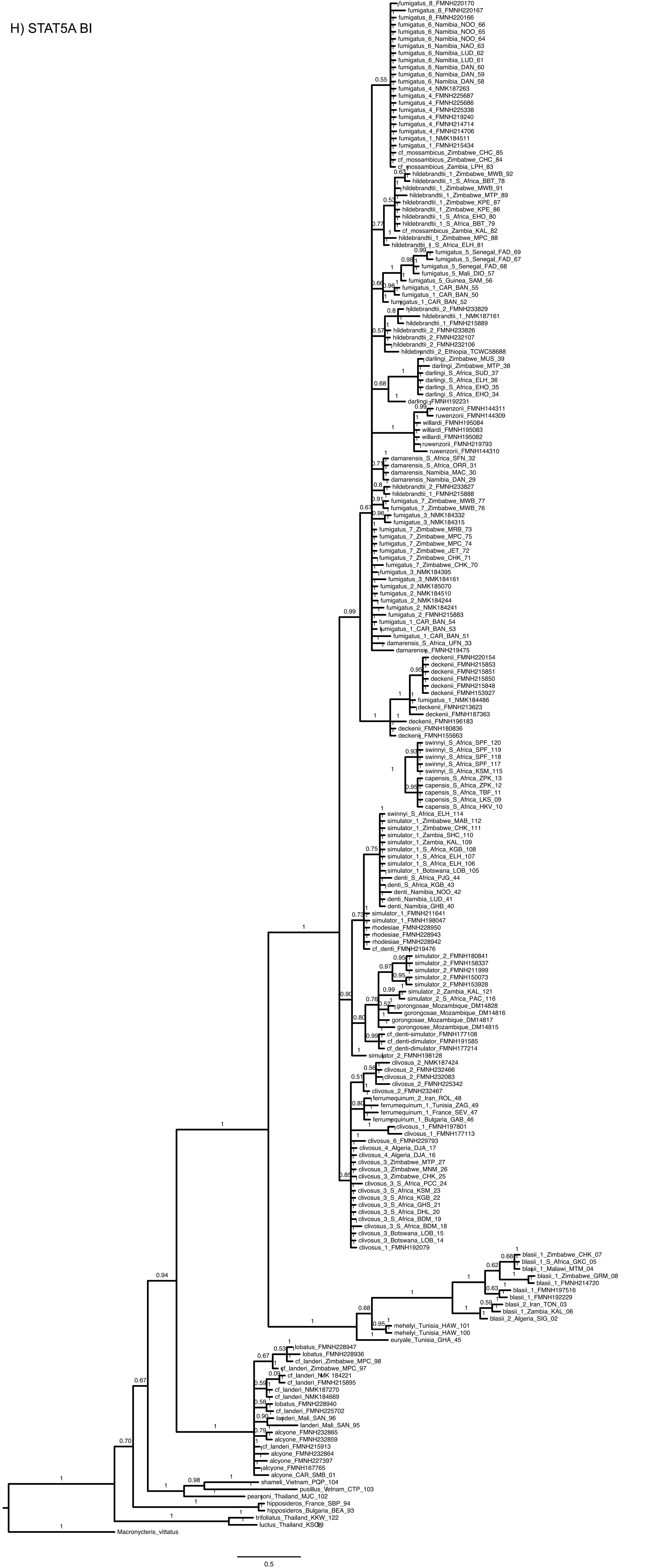

Supplement: Supplementary file 6 — Maximum likelihood gene trees inferred for nuclear introns using IQ-TREE (A–D) and Bayesian gene trees inferred for nuclear introns using MRBAYES (E–H). Nodal support is indicated above branches. Museum acronyms are defined in Additional file 1. (PDF 1980 kb) [file 12862_2019_1485_MOESM6_ESM.pdf]
