# Supplementary material for: Molecular phylogenetics of the African horseshoe bats (Chiroptera: Rhinolophidae): expanded geographic and taxonomic sampling of the Afrotropics
Source: BMC Evol Biol. 2019 Aug 22;19:166. doi: 10.1186/s12862-019-1485-1 (PMC6704657; doi:10.1186/s12862-019-1485-1)

Additional file 5. Maximum likelihood gene tree inferred for cyt-*b* using IQ-TREE that includes two sequences of *R. gorongosae* deposited in GenBank (Taylor et al. 2018; indicated by red font) and five individuals newly sequenced for cyt-*b* in this study (indicated by blue font). DM14815 is included twice in the tree (GenBank sequence and new sequence from this study). Bootstrap support is indicated above branches. Nodes with < 50% bootstrap support not labled.

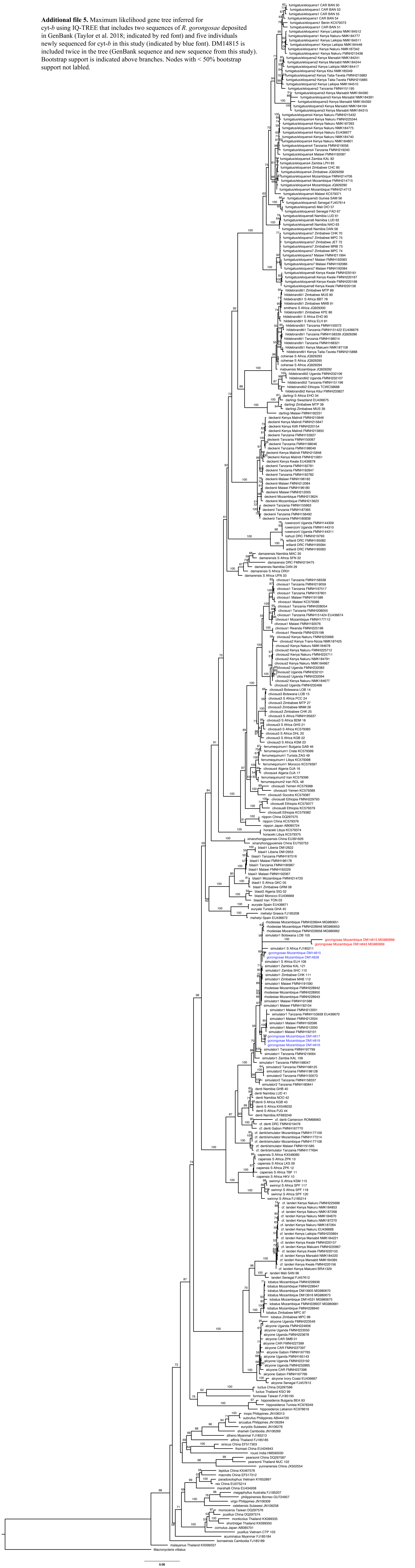

Supplement: Supplementary file 5 — Maximum likelihood gene tree inferred for cyt-b using IQ-TREE that includes two sequences of R. gorongosae deposited in GenBank [12; indicated by red font] and five specimens newly sequenced for cyt-b in this study (indicated by blue font). DM14815 is included twice in the tree (both the GenBank sequence and a newly generated sequence from this study). Nodal support is indicated above branches. Museum acronyms are defined in Additional file 1. (PDF 533 kb) [file 12862_2019_1485_MOESM5_ESM.pdf]
